# Genome-wide association study of a Guinea grass (*Megathyrsus maximus*) diversity panel reveals the genetic basis of agronomic and nutritional traits

**DOI:** 10.1186/s12870-025-08007-2

**Published:** 2025-12-30

**Authors:** Lina M. López-Contreras, Kate E. Denning-James, Juliana Carvajal-Tapia, Jacobo Arango, Daniel M. Villegas, Rosa N. Jauregui, Peter Wenzl, Jose J. De Vega, Monica Carvajal-Yepes

**Affiliations:** 1 International Center for Tropical Agriculture (CIAT), A. A, 6713, Km 17 Recta Cali-Palmira, Palmira, Valle del Cauca Colombia; 2 Earlham Institute, Norwich Research Park, Norwich, UK; 3 Faculty of Agricultural Sciences, NUTRIFACA Research group, Universidad del Cauca, Popayán, Colombia

**Keywords:** *Megathyrsus maximus*, *Panicum maximum*, GWAS, Marker-trait association, Forage breeding, Nitrogen metabolism, Lignin biosynthesis, Yield-related traits, Tropical forage crops

## Abstract

Guinea grass (*Megathyrsus maximus*) is a common tropical forage crop valued for its high biomass yields, nutritional quality, and adaptability to diverse abiotic stresses. However, the genetic basis of its key agronomic traits remains poorly understood. In this study, we conducted genome-wide association studies (GWAS) using whole genome sequencing (WGS) data from 124 diverse *M. maximus* genebank accessions, yielding a high-density single-nucleotide polymorphism (SNP) data set of 1,261,156 SNPs after mapping to a related reference genome. Population structure analysis revealed three major genetic subgroups within the collection. Using three complementary GWAS models (Bayesian-information and Linkage-disequilibrium Iteratively Incorporating Knowledge (BLINK), the Fixed and Random Model Circulating Polynomial Unification (FarmCPU), and the Multiple Loci Mixed Model (MLMM)), we identified 25 significant marker-trait associations (MTAs) spanning three major trait categories: (i) phenological and plant-architecture; (ii) nutritional and digestibility; and (iii) productivity and nitrogen-use traits, evaluated under wet-season, dry-season, and greenhouse conditions. Several MTAs were associated with genes related to plant growth and development, lignin biosynthesis, and nitrogen-cycling (including nitrogen uptake and biological nitrification inhibition). Notably, two SNPs were pleiotropic, with one associated with both nitrogen uptake and shoot biomass production, and another shared between crude protein and in vitro dry matter digestibility. Although a complete reference genome is not yet available for *M. maximus*, our results provide valuable information for future marker validation and breeding. This study highlights the potential of GWAS as a powerful tool for trait dissection and genetic improvement in tropical forage crops. The research represents a step forward in developing more resilient and productive *M. maximus* cultivars.

**Supplementary Information:**

The online version contains supplementary material available at 10.1186/s12870-025-08007-2.

## Background

Forages play a crucial role in supporting livestock production and enhancing the nutritional value and sustainability of meat and dairy products consumed by humans [1]. In addition, they provide important ecosystem services as cover crops, such as improving soil health, reducing erosion, and enhancing biodiversity [2]. However, increasing population and agricultural land pressures limit the availability of high-quality forages, which negatively affects livestock productivity [3]. Protein-rich and low-fiber grasses with reduced neutral detergent fiber (NDF) and lignin content lower feed-retention time in the rumen, improve digestibility, increase weight gain, and reduce enteric methane emissions [4, 5]. This dual role—supporting livestock health and productivity while providing ecosystem services under challenging environmental conditions—underscores the critical importance of forages in sustainable agriculture and human nutrition.

*Megathyrsus maximus* (Jacq.) B.K. Simon & S.W.L. Jacobs (syn. *Panicum maximum* Jacq.), commonly known as Guinea grass, is a highly valued grass forage species widely used in tropical and subtropical regions worldwide [6, 7]. It is favored for pasture establishment, high productivity, and silage [8, 9]. Guinea grass genotypes exhibit resilience under the seasonal wet and dry periods typical of tropical climates [7]. Its high nutritive value, perennial growth habit, beneficial interactions with the rhizosphere, resistance to drought and salinity, and invasive potential further contribute to its successful integration into forage-livestock systems [6]. Originally introduced as animal feed, Guinea grass is recognized for its adaptability, hardiness, and high dry matter yield (DMY) [10]. The species reproduces both sexually and through apomixis [6] and is predominantly autotetraploid, with its origins in Africa [7, 11]. Livestock farmers can maintain forage quality and digestibility through effective management practices, such as rotational grazing or regular cutting. Despite its high productivity, Guinea grass may require additional fertilization in nutrient-poor soils to sustain growth and nutritional quality. Under drought stress, nitrogen (N) uptake is reduced, further limiting its performance [12]. Nevertheless, the CIAT Guinea grass germplasm collection includes accessions that are tolerant to diverse climatic conditions. For instance, the commercial cultivar Mombasa and accession 6982 have demonstrated resilience, persistence, and stable performance under the adverse edaphoclimatic conditions of the dry tropics [7].

Beyond their adaptability, tropical forages such as *M. maximus* are increasingly recognized not only for their role in animal nutrition but also for their environmental benefits. Breeding programs for tropical grasses (*Urochloa* spp., *Megathyrsus* spp.) have successfully targeted traits such as biomass productivity, digestibility, and biological nitrification inhibition (BNI), which reduces soil nitrous oxide (N_2_O) emissions, a highly potent greenhouse gas [13]. These advances highlight the untapped potential of *M. maximus* for similar improvements.

Current efforts in tropical forages breeding focus on increasing biomass yields and nutritional quality, as well as improving tolerance to biotic and abiotic stressors. Association mapping has greatly advanced our understanding of the genetic architecture underlying complex traits in forage species. In particular, the identification of DNA markers and their associated genes is valuable for the genetic improvement of forage quality [14]. Genome-wide association studies (GWAS) have already been conducted for traits such as crude protein (CP), biomass yield, NDF, and acid detergent fiber (ADF) in various temperate forage species, including *Sorghum bicolor*,* Medicago sativa*, and *Cenchrus ciliaris* [14–16]. Many of the associated markers have been identified in genomic regions containing genes involved in biochemical pathways related to yield, stress response, and forage quality [16]. The application of high-throughput methods for detecting markers and genes underlying these quality traits is essential for accelerating the genetic improvement of tropical forages [14].

Despite progress in genetic studies related to other forage crops, there has been limited focus on *M. maximus*. Globally, only a few Guinea grass breeding programs exist, and these began just three to four decades ago, mostly within public institutions [17–19]. Additionally, the species’ polyploidy, high levels of heterozygosity, and the absence of a reference genome hinder detailed genetic analysis. This highlights the need for developing more precise genomic characterization and improved selection models [20]. Previous research on the International Center for Tropical Agriculture (CIAT) Guinea grass germplasm collection has identified accessions with promising traits for forage production under tropical conditions [7] and for BNI [13].

The genomic characterization of CIAT’s germplasm collection is of critical importance for breeding programs, as it enables the use of the genetic diversity to address current and future challenges, including climate change, pests and diseases, and increasing production needs. In this study, we tested the hypothesis that genomic regions associated with agronomic and nutritional quality traits in *M. maximus* can be identified using a reference genome from a related species. Such identification is expected to enhance the effective utilization of genetic resources and provide a valuable foundation for breeding programs. Using 124 accessions of *M. maximus*, we identified marker-trait associations (MTAs) across 13 chromosomes of *Urochloa decumbens*, related to agronomic, nutritional quality, and yield traits assessed under field and greenhouse conditions. These findings represent a significant advance in understanding how key traits are mediated in forage crops. They support breeding efforts by facilitating the development of more resilient forage varieties suited to meet the growing demand for animal-sourced food products. These advances can help crop-improvement specialists overcome the increased production challenges posed by climate change.

## Methods

### Source of the phenotypic data

The accession panel used in this study comprised 124 accessions of *Megathyrsus maximus* (syn. *Panicum maximum*) conserved in the CIAT genebank, with phenotypic data obtained from previous evaluations conducted by CIAT’s Tropical Forages program [7, 13, 21]. In total, 19 variables (“Var”), representing 11 agronomic traits, were analyzed across three evaluation environments: wet in-field season, dry in-field season, and greenhouse conditions (Supplementary Table 1). For clarity in presenting the results, the evaluated traits were grouped into three categories: (i) Phenological and plant-architecture traits (for flowering and plant height (PH)); (ii) Nutritional quality traits (including crude protein (CP), in vitro dry matter digestibility (IVDMD), ADF, and NDF; and (iii) Biomass production and N-use traits (N uptake, nitrification rate (NR), shoot biomass production (SBP), green forage weight (GFW), and DMY). Throughout the manuscript, we use the label “Var” to refer to each phenotypic variable within each trait, with variable numbering assigned in consecutive order. As a result, some traits are presented by one or two variables (e.g., flowering percentage), whereas others have multiple variables (e.g., PH or DMY) because they were measured across different seasons or evaluation periods. Throughout the manuscript, we use the label “Var” to refer to each phenotypic variable within each trait, with variable numbering assigned in consecutive order. As a result, some traits are presented by one or two variables (e.g., flowering percentage), whereas others have multiple variables (e.g., PH or DMY) because they were measured across different seasons or evaluation periods.

Phenological and plant-architecture traits: Flowering percentage was evaluated through two variables: Var1, the average flowering percentage measured during the dry season, and Var2, the Best Linear Unbiased Estimators (BLUEs)-derived flowering percentage measured during the wet season. Plant height (PH) was represented by four variables: Var3, the average PH collected during the wet season; Var4, the average PH collected during the dry season; Var5, the BLUEs PH value from wet-season evaluations; and Var6, the BLUP-estimated PH.

Nutritional quality traits: CP content was captured through Var7 and Var8, representing average CP percentages measured during the dry and wet seasons, respectively. IVDMD was assessed in two wet-season evaluations, with Var9 representing the average IVDMD percentage collected eight weeks after cutting, and Var10 representing the average collected four weeks after cutting. Fiber-related traits, evaluated in a subset of 117 accessions, included Var11 (ADF) and Var12 (NDF), both measured during the wet season.

Biomass production and N-use traits: A greenhouse experiment with 115 accessions provided Var13 (average N uptake (g N/ pot)); Var14, average soil NR, used to quantify BNI expressed as mg NO₃⁻ per kg soil per day; and Var15, average SBP expressed in ton/ha. Field biomass traits included Var16, the BLUEs GFW (GFW, ton/ha) measured during the dry season, and Var17–Var19, the BLUEs dry matter yield (DMY) values collected across three distinct field conditions or evaluation periods.

Field trials were conducted in Valle del Patía, Colombia, where the mean temperature is 27.9 °C and rainfall follows a bimodal pattern, with an annual mean precipitation of 1.414 mm. The local soil is classified as a medium-fertility Mollisol, with chemical properties described previously by Carvajal-Tapia et al. [7, 21]. The experiment began in December 2015, with evaluations performed throughout 2016 and 2017, five during the wet season and two during the dry season. All accessions were subjected to a standardized cutting (sc) at 30 cm height before each evaluation [13, 21]. 

Greenhouse trials followed methodologies described by Villegas et al. [13] and targeted traits associated with N cycling and BNI, which are of increasing importance for tropical forages improvement.

### Treatment of phenotypic data

Best Linear Unbiased Estimators (BLUEs) were calculated for multiple traits, including flowering percentage for Var2; PH for Var5; GFW for Var16, and DMY for Var17 and Var18. The estimates were obtained using the *polyqtlR* package in R (version 4.3.2) [22], based on a linear mixed model defined as:

$$\:Yij=\mu\:+\tau\:i+\beta\:j+\epsilon\:ij$$

where $\:Yij$ is the observed phenotypic value of genotype $\:i$ in block $\:j$, $\:\mu\:$ is the overall mean, $\:\tau\:i$ is the fixed effect of the $\:i\:$genotype (accession), βj is the random effect of the $\:j$ block, and $\:\epsilon\:ij$ is the residual error. In this model, genotypes (accessions) were treated as fixed effects to estimate genotype-specific performance, while blocks were modeled as random effects to account for environmental variability across replicates. This approach provided unbiased genotype estimates across the three blocks evaluated for each trait [23].

Best Linear Unbiased Predictors (BLUPs) used in the analysis were taken from Carvajal-Tapia et al. [7]. Descriptive statistics were calculated for all variables. The distribution of each phenotypic variable (Var1–Var19) was assessed using the Shapiro–Wilk test to evaluate normality prior to GWAS analysis. Data visualization was performed using R software (http://www.R-project.org/), including frequency distribution histograms, Manhattan plots, and quantile-quantile plots.

### Sequencing and genotyping

All the samples’ genomic DNA was isolated from the leaf tissue of plants growing either in a field trial or a CIAT greenhouse. For each germplasm accession, three leaf pieces of 0.5 cm^2^ were sampled in 2 mL tubes, frozen in liquid N, and subjected to bead beating for 10 min. Then, 700 µL of extraction buffer were added, containing: (i) 100 mM Tris-HCl pH 8.0; (ii) 1.4 mM NaCl; (iii) 20 mM Ethylene diamine-tetra-acetic acid (EDTA) pH 8.0; (iv) 1% polyethylene glycol (PEG) 6000; (v) 2% mixed alkyl-trimethylammonium bromide (MATAB); and (vi) 0.5% sodium bisulfite. The samples were incubated for 30 min at 74 °C [24]. A proportion of 1:1 of Chloroform isoamyl alcohol 24:1 was added and the samples were centrifuged at 13,000 rpm for 15 min. An equal volume of supernatant was transferred to a new tube and DNA was precipitated by adding 330 µL of 2-propanol, incubated at -20 °C for 2 hours, and centrifuged as before. The DNA pellet was washed with 200 µL of 80% ethanol, followed by centrifugation and vacuum drying. DNA was re-suspended in TE Buffer/RNase A 10:1 (v/v) and incubated at 37 °C for 30 min. DNA was quantified with a NanoDrop 2000 spectrophotometer (Thermo Scientific, Massachusetts, USA) and visualized on a 0.8% agarose gel.

The genotypic data were obtained through whole genome sequencing (WGS) using Illumina short reads, performed at the Earlham Institute (Norwich, UK). The sequenced population consisted of 128 accessions of Guinea grass from CIAT’s germplasm collection. After quality filtering, 124 of the accessions were retained for analysis, while two accessions (CIAT6951 and CIAT16062) and two cultivars (*Naturalized*, and *Sabanera*) were excluded from further analysis due to sequencing coverage quality.

Raw sequencing reads were assessed using the FastQC package [25] and trimmed with TrimGalore (v. 0.5.0) [26] to remove adapters and poor-quality reads. SAMtools were used to filter primary alignments and to sort them. The FASTQ files were aligned to two different reference genomes, *Setaria viridis* and *Urochloa decumbens* cv. Basilisk genome, using the Burrows-Wheeler Aligner maximum exact match algorithm (BWA-MEM) [27]. The resulting Binary Alignment Map (BAM) files were then indexed against the respective reference genome. *Urochloa decumbens* cv. Basilisk [28] and *Setaria viridis* v1.1 [29] reference genomes were downloaded at the National Center for Biotechnology Information (NCBI) and the United States Department of Energy’s Joint Genome Institute (JGI) Phytozome online portal, respectively. Polymerase chain reaction (PCR) duplicates were annotated with Picard Tools [30]. Single-nucleotide polymorphism calling was implemented using the Genome Analysis Toolkit (GATK)’s [31] standard DNA pipeline, and the variants were hard-filtered with BCFtools (v.1.12) using the thresholds recommended by the GATK guidelines [32].

According to the best-hit reads mapped to the two reference genomes, we decided to use *Urochloa decumbens* cv. Basilisk genome. The initial variant calling generated approximately 30 million SNPs, which were subsequently filtered based on SNP quality and coverage. The final dataset consisted of a matrix with 1,261,156 SNPs. The applied filters included: (i) a minimum depth of coverage (> 5); (ii) a minor allele frequency (MAF) threshold of 0.02; (iii) a quality filter (removing variants with a call quality score below 30); and (iv) a restriction to biallelic SNPs (a maximum of two alleles per SNP).

### Population structure

A pairwise genetic distance matrix (1-IBS, identity by state) was calculated using 1,261,156 SNP markers with the *snpgdsIBS* function from the *SNPRelate* package (v1.32.2) in R (v4.2.1) [33]. The distance matrix was used for agglomerative clustering analysis using the minimum variance method (Ward.D2), implemented through the *hclust* function in the *stats* R package (v4.2.1) [34]. The optimal number of ancestral populations (K) was estimated using the *snfm* function from the *LEA* package (v3.19.8) [35]. Visualizations were generated using the *ggplot2* package (v3.5.1) [36].

### Linkage disequilibrium analysis

Linkage disequilibrium (LD) decay was evaluated using the filtered SNP dataset (1,261,156 SNPs). Linkage disequilibrium was calculated in TASSEL v5 using a sliding window of 10 SNPs and setting heterozygous SNPs as missing. Pairwise LD was estimated using the r² coefficient, r² values were plotted against physical distance. Linkage disequilibrium decay was summarized by fitting a nonlinear regression curve and defining the decay distance as the point at which r² dropped below 0.2.

### Genome-wide association study

A genome-wide association study (GWAS) was conducted using 19 phenotypic variables, with data collected for each accession. A haplotype file of 1,261,156 SNPs was generated for the analysis. Three mixed linear models were evaluated: the Bayesian-information and Linkage-disequilibrium Iteratively Incorporating Knowledge (BLINK) model [37]; the Fixed and Random Model Circulating Polynomial Unification (FarmCPU) [38], and the Multiple Loci Mixed Model (MLMM) [39]. These models were implemented using the GAPIT (Genome Association and Prediction Integrated Tool) package version 3 in R [40]. A Bonferroni correction was applied with a significance threshold of 0.05. Significant associations were visualized using Manhattan plots, where clusters of markers with -log10(p-value) scores 5 > formed distinctive “skyscraper” patterns. Quantile–quantile (QQ) plots were generated for each trait–model combination to evaluate the performance of the BLINK, FarmCPU, and MLMM models in controlling for population structure and relatedness. Markers identified as significant in at least two models were highlighted and annotated.

The physical positions of significant SNP associations were mapped to the *Urochloa decumbens* cv. Basilisk CIAT 606 (2n = 4x = 36) reference genome [28]. Additionally, QQ plots were used to compare observed and expected p-values, helping to evaluate each model’s performance in controlling Type I errors due to population structure and kinship relationships [41].

### Functional annotation and identification of candidate genes

The *Urochloa decumbens* cv. Basilisk reference genome was used for gene annotation. Candidate genes were identified based on their proximity to the peak SNP markers using a 40 kb flanking region (20 kb upstream and 20 kb downstream) to capture nearby regulatory elements and gene functions [42]. To visualize the SNP positions and assess their genomic context, the Integrative Genomics Viewer (IGV; https://software.broadinstitute.org/software/igv/) was used. Single-nucleotide polymorphisms (SNPs) classified as “high-impact” mutations by *SnpEff* (version 1.9.6) [43, 44] within a 40 kb flanking region were identified, as these are predicted to significantly affect gene function.

## Results

### Diversity panel of *M. maximus* accessions and phenotypic data description

Substantial phenotypic variation was observed among the 124 *M. maximus* accessions evaluated for the 19 variables representing 11 agronomic and nutritional traits under three contrasting environmental conditions: wet season, dry season, and greenhouse trials **(**Table 1**)**. Descriptive statistics and histograms confirmed wide variability for most traits (Table 2, Supplementary Fig. 1).

**Table 1:** Phenotypic variables evaluated in the panel of *Megathyrsus maximus* accessions analyzed (sc*: standardized cutting). Source: 2020: [13]; 2022: [7], and this study

Trait	Description	Vars	Season	Evaluation period	Weeks after sc*	Source	Units	Used Data	*N*
Flowering	The percentage of flowering was estimated based on visual observations of the experimental plot (4 m²) using a scale of 0–100%.	Var1	Dry	4 May to 1 June 2017 28 Dec. 2016 to 9 Feb. 2017 11 Feb. to 23 Mar. 2017 4 May to 29 June 2017 2 May to 1 Junio 2017	4 6 8	2022	%	Average	124
		Var2	Wet	4 May to 29 June 2017	8	2022	%	BLUEs	124
Plant Height (PH)	Height was measured according to Toledo and Schultze-Kraft (1982) using a 1m^2^ quadrant.	Var3	Wet	N/A	N/A	2022	cm	Average	124
		Var4	Dry	N/A	N/A	2022	cm	Average	124
		Var5	Wet	22 June to 2 Aug. 2016	6	2022	cm	BLUEs	124
		Var6	N/A	N/A	N/A	2022	cm	BLUP	124
Crude Protein (CP)	Crude protein content was estimated by multiplying the total nitrogen concentration in shoot tissue by a conversion factor of 6.25 (AOAC, 2002).	Var7	Dry	30 June to 24 Aug. 2017	8	2022	%	Average	124
		Var8	Wet	4 May to 1 June 2017	4	this study	%	Average	124
In Vitro Dry Matter Digestibility (IVDMD)	Forage digestibility was evaluated by incubating ground samples with rumen fluid under controlled laboratory conditions to simulate the digestive process. Higher IVDMD values indicate superior forage quality.	Var9	Wet	4 May to 29 June 2017	8	this study	%	Average	124
		Var10	Wet	4 May to 1 June 2017	4	this study	%	Average	124
Acid Detergent Fiber (ADF)	Acid detergent fiber contains cellulose and lignin, the least digestible components of the plant. Lower ADF values indicate higher digestibility and greater energy availability.	Var11	Wet	24 Mar. to 4 May 2017	6	2022	%	Average	117
Neutral Detergent Fiber (NDF)	Neutral detergent fiber represents the total cell wall composition of the forage, including hemicellulose, cellulose, and lignin.	Var12	Wet	24 Mar. to 4 May 2017	6	2022	%	Average	117
N Uptake	Nitrogen uptake was calculated by multiplying the nitrogen concentration by the yield of aboveground biomass.	Var13	Greenhouse	N/A	N/A	2020	gr N/pot	Average	115
Nitrification Rate (NR)	Nitrification rate was calculated as the slope of a linear regression between concentrations of NO3- and incubation time.	Var14	Greenhouse	N/A	N/A	2020	mg NO₃⁻ kg⁻¹ soil day⁻¹	Average	115
Shoot Biomass Production (SBP)	Aboveground biomass was trimmed to 15 cm to standardize the trial, and shoots were collected after 35 days of regrowth, then oven-dried at 60 °C for 72 h.	Var15	Greenhouse	N/A	N/A	2020	gr dry matter/pot	Average	115
Green Forage Weight (GFW)	The foliage was obtained after cutting at 30 cm above ground level from each plot.	Var16	Dry	30 June to 24 Aug. 2017	8	2022	Ton/ha	BLUEs	124
Dry Matter Yield (DMY)	Green forage samples (200 g) were collected from each plot after cutting at 30 cm height and oven-dried at 60–70 °C until constant weight to estimate DMY.	Var17	Wet	24 Mar. to 4 May 2017	6	2022	Ton/ha	BLUEs	124
		Var18	Dry	30 June to 24 Aug. 2017	8	2022	Ton/ha	BLUEs	124
		Var19	N/A	N/A	N/A	2022	Ton/ha	BLUEs	124

Phenological and plant-architecture traits: Flowering percentages (Var1, Var2) showed the greatest dispersion, ranging from 0 to 100%. Flowering based on BLUEs during the wet season (Var2), had a higher mean (62.95%) and greater variability (SD = 37.64). Plant height (PH) was consistently greater in the wet season (Var3: mean = 119.07 cm) than in the dry season (Var4: mean = 80.10 cm), reflecting environmental influence on vegetative growth. Moderate variation was observed for nutritional traits. Crude protein (Var7 and Var8) ranged from 4.95% to 17.47%, with mean values of 7.37 (dry season) and 12.94% (wet season). In vitro dry-matter-digestibility (IVDMD, Var9 and Var10) also varied moderately, with mean values between 56.14% and 60.74% across seasons. Fiber-related traits showed relatively high averages; ADF (Var11) averaged 40.56%, while NDF (Var12) averaged 64.53%. For biomass and N-cycling traits, the traits from the greenhouse experiment showed a wide variation, with N uptake (Var13) ranging from 4.59 to 10.71 g N/pot; NR (Var14) averaging 6.58 mg NO₃⁻ kg⁻¹ soil day⁻¹; and SBP (Var15) ranging from 6.03 to 28.07 g dry matter/pot. Under field conditions, GFW (Var16) varied from 1.77 to 17.37 t ha⁻¹ and DMY (Var17-Var19) ranged from 0.77 to 7.33 t ha⁻¹. The Shapiro–Wilk tests indicated that 8 of the 19 variables were normally distributed (*p* > 0.05), whereas the remaining traits deviated from normality (Table 2).

**Table 2:** Descriptive statistics for the 19 phenotypic variables evaluated in *Megathyrsus maximus*. The table includes mean, standard deviation (SD), minimum, and maximum values, along with Shapiro–Wilk p-values and a normality classification (Normal for *p* > 0.05; not normal for *p* < 0.05)

Trait	Variables	Min	Max	Median	Mean	SD	Shapiro-Wilk *p*-value	Normality
Flowering	var1	0.00	100.00	63.77	56.33	26.14	1.19 × 10⁻⁷	Not normal
	var2	0.00	101.79	80.00	62.95	37.64	1.46 × 10⁻¹⁰	Not normal
Plant Height (PH)	var3	61.13	149.40	120.70	119.07	16.17	1.50 × 10⁻³	Not normal
	var4	48.67	109.67	82.19	80.10	15.65	4.50 × 10⁻³	Not normal
	var5	74.99	163.33	132.58	130.72	18.88	1.25 × 10⁻⁴	Not normal
	var6	65.03	134.29	111.58	109.24	13.67	5.58 × 10⁻⁴	Not normal
Crude Protein (CP)	var7	4.95	10.52	7.35	7.37	1.00	3.57 × 10⁻¹	Normal
	var8	10.02	17.47	13.03	12.94	1.26	1.79 × 10⁻¹	Normal
In Vitro Dry Matter Digestibility (IVDMD)	var9	48.94	61.92	56.38	56.14	2.77	2.71 × 10⁻¹	Normal
	var10	53.53	65.97	60.96	60.74	1.99	9.53 × 10⁻³	Not normal
Acid Detergent Fiber (ADF)	var11	35.05	48.01	40.34	40.56	2.84	7.16 × 10⁻²	Normal
Neutral Detergent Fiber (NDF)	var12	59.05	70.68	64.21	64.53	2.54	7.90 × 10⁻²	Normal
N uptake	var13	62.80	235.41	145.31	149.62	31.98	1.34 × 10⁻¹	Normal
Nitrification Rate (NR)	var14	4.59	10.71	6.44	6.58	1.02	1.61 × 10⁻⁷	Not normal
Shoot Biomass Production (SBP)	var15	6.03	28.07	17.07	17.47	3.75	1.04 × 10⁻²	Not normal
Green Forage Weight (GFW)	var16	1.77	17.37	8.32	8.50	2.55	2.10 × 10⁻³	Not normal
Dry Matter Yield (DMY)	var17	3.08	11.95	5.58	5.78	1.55	4.27 × 10⁻⁵	Not normal
	var18	0.77	6.09	3.23	3.28	0.87	2.78 × 10⁻¹	Normal
	var19	2.38	7.33	4.47	4.51	0.83	3.09 × 10⁻¹	Normal

**Fig. 1:**
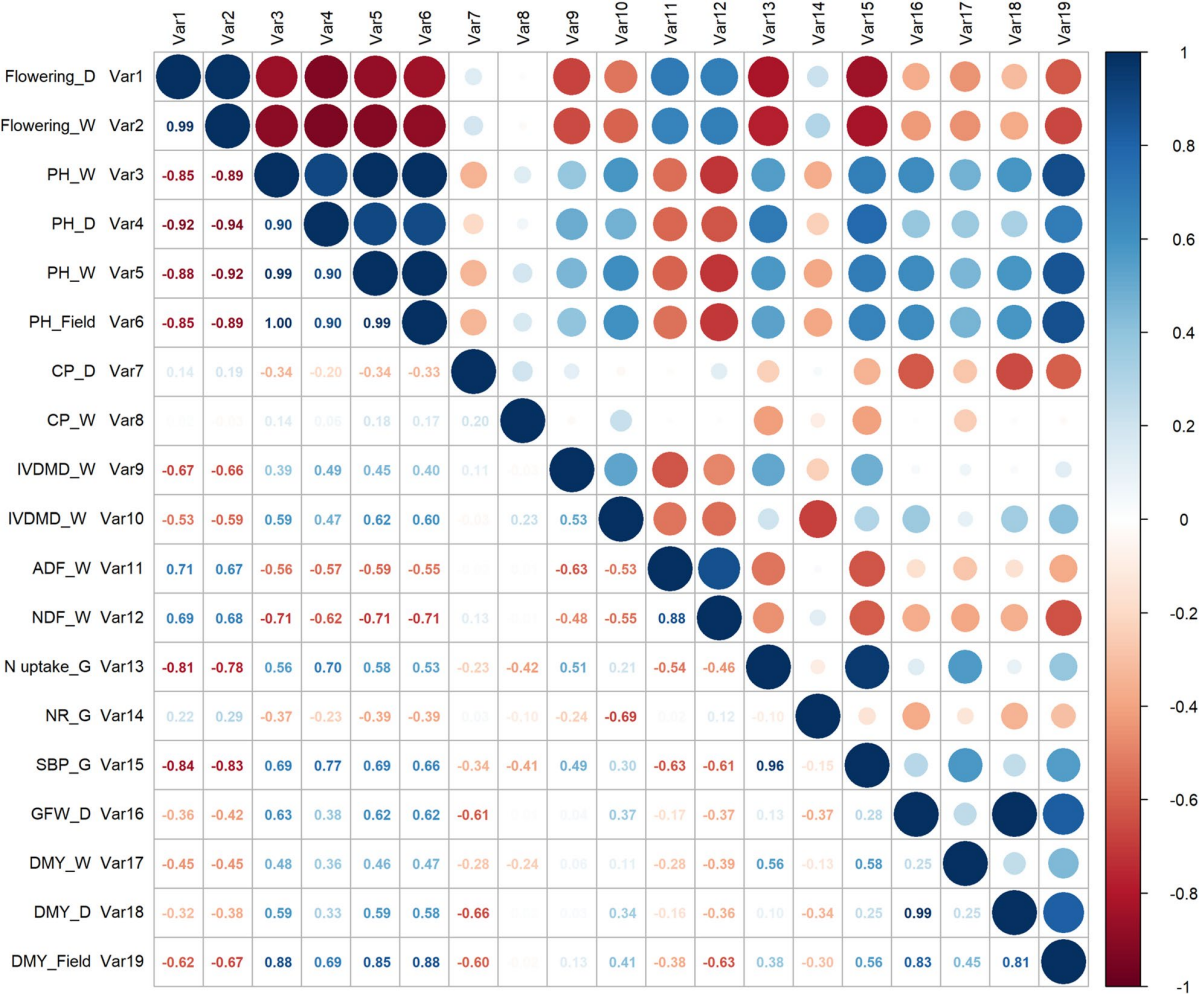
Phenotypic correlations among variables. Variables related to growth, nutritional quality, yield, nitrogen uptake, and nitrification rate under dry (D), wet (W), and greenhouse (G) conditions. Field data correspond to BLUPs (Best Linear Unbiased Predictors) estimated for each variable

A correlation analysis revealed distinct relationships among the 19 evaluated variables (|r| > 0.7) **(**Fig. 1**)**. Flowering variables (Var1 and Var2) were strongly negatively correlated with all plant height variables (Var3-Var6, *r* = -0.85 to -0.94), and also with IVDMD (Var9 and Var10, *r* = -0.62 and − 0.67, respectively); SBP (Var15, *r* = -0.84), and DMY (Var19, *r* = -0.62 and − 0.67). In contrast, flowering showed positive correlation with ADF (Var11, *r* = 0.71/0.67) and NDF (Var12, *r* = 0.69). Plant height (PH) variables were almost perfectly correlated with each other (*r* = 0.90 to 1.00), and positively correlated with N uptake (Var13, *r* = 0.53 to 0.70) and DMY (Var19, *r* = 0.69 to 0.88). Nitrogen (N) uptake (Var13) also showed a strong correlation with SBP (Var15, *r* = 0.96). For nutritional traits, IVDMD (Var9 and Var10) was negatively correlated with ADF (Var11, *r* = − 0.53 to 0.63) and NDF (Var12, *r* = 0.48 to 0.55). Finally, for biomass traits, GFW (Var16) was positively correlated with DMY (Var18 and Var19, *r* = 0.83 and 0.99, respectively).

### Population structure

**Fig. 2:**
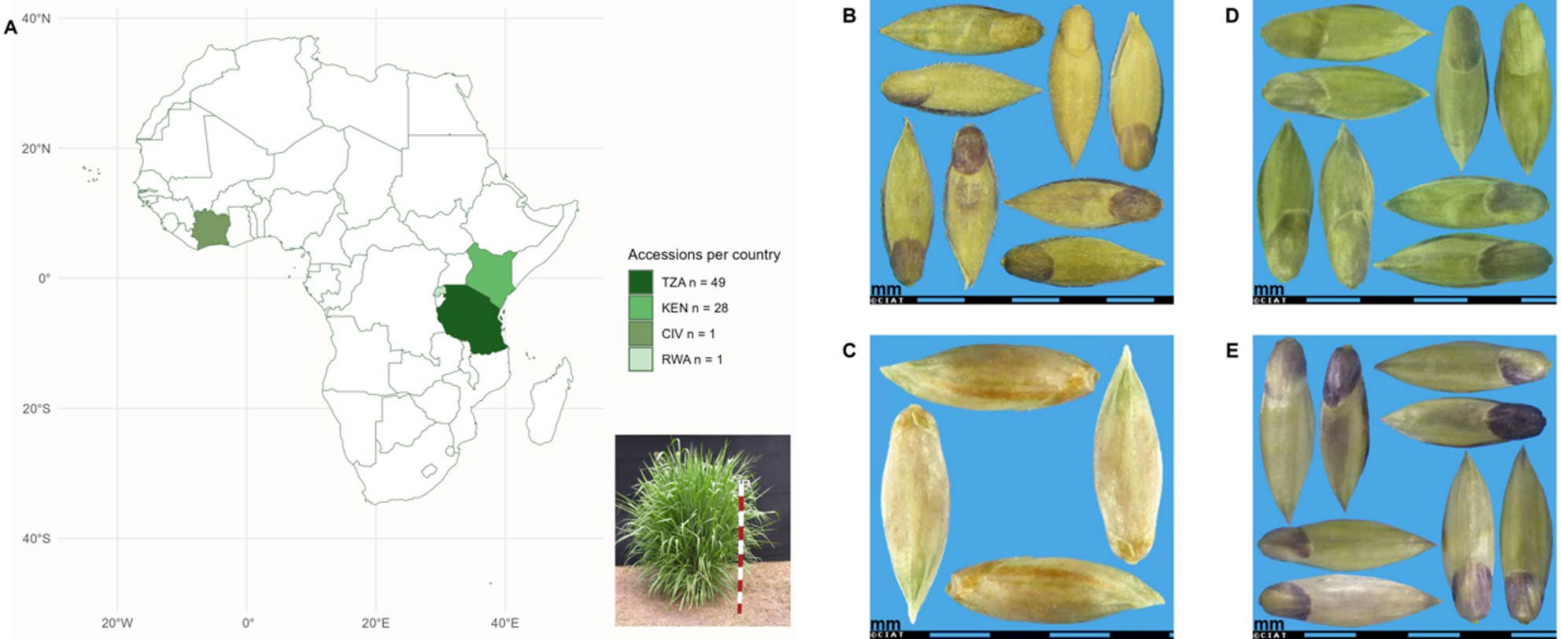
Geographic origin of 79 *Megathyrsus maximus* accessions (**A**); Seed images of selected accessions: CIAT622 from Kenya (KEN) (**B**), CIAT6872 from Côte d’Ivoire (CIV) (**C**), CIAT16049 from Tanzania (TZA) (**D**), and CIAT26360 from Rwanda (RWA) (**E**)

Whole genome sequencing (WGS) enabled the successful genotyping of all the accessions, providing a comprehensive SNP dataset for downstream analyses. The 124 *M. maximus* accessions analyzed showed a predominance of material originating from African countries, with seeds exhibiting uniform shapes and minor variation in color (Figs. 2A-E). The largest number of accessions was collected in Tanzania (49), followed by Kenya (28), while Rwanda and Côte d’Ivoire were each represented by a single accession (Fig. 2A). The origin of the remaining 45 accessions is unknown. Only four accessions correspond to improved breeding lines: CIAT26900 (Vencedor), CIAT6299, and two developed in Tanzania, CIAT6962 (Momobaca) and CIAT16031.

**Fig. 3:**
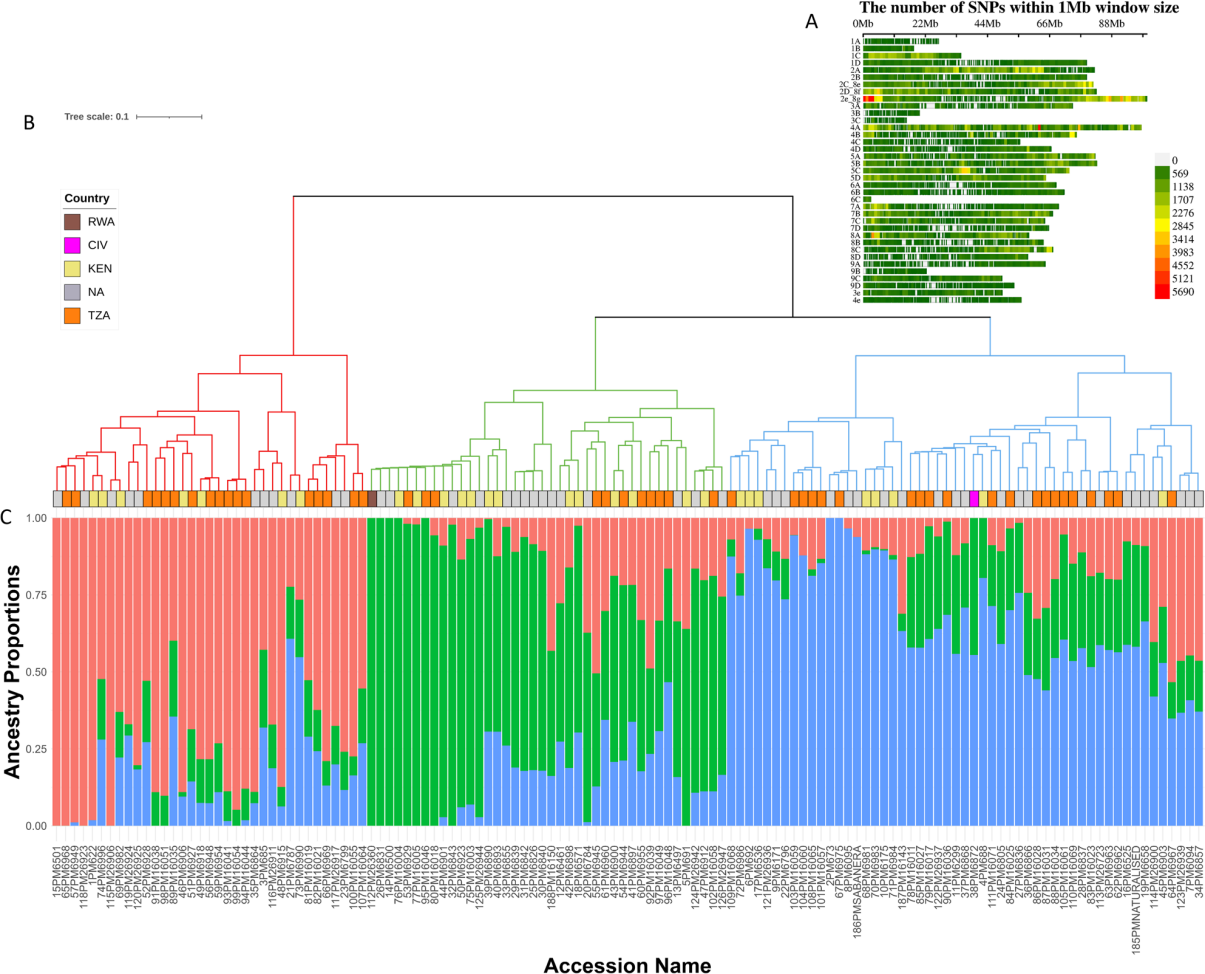
Population structure of 124 *Megathyrsus maximus* accessions based on whole genome sequencing (WGS) data. **A** SNP density plot showing the distribution of 1,261,156 SNPs across the 39 chromosomes of the *Urochloa decumbens* cv. Basilisk reference genome. **B** Hierarchical clustering of accessions based on the Identity-by-State (IBS) distance matrix using the Ward.D2 linkage method. **C** Ancestry coefficients from sNMF analysis at *K* = 3, shown as a stacked bar plot for individual accessions. Colors indicate membership proportions in the three inferred genetic clusters

Hierarchical clustering based on the Identity-by-State (IBS) distance matrix revealed three distinct genetic groups **(**Fig. 3A). This structure was further supported by sparse non-negative matrix factorization (sNMF) analysis, which identified three clusters based on individual ancestry coefficients **(**Fig. 3B). The assignment of accessions to each cluster was consistent across methods and no clear genetic structure was observed with respect to the country of origin.

**Fig. 4:**
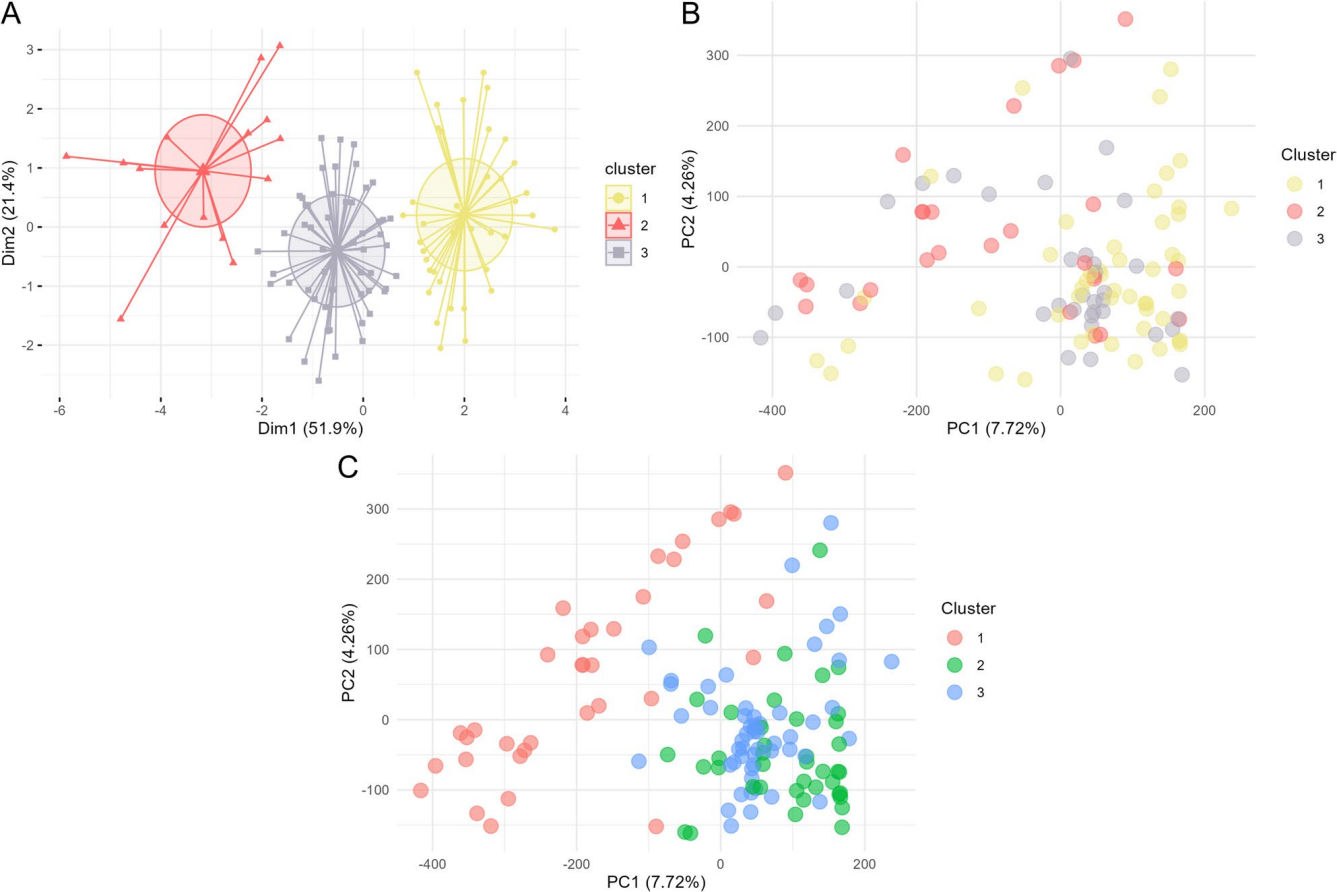
K-means clustering and PCA-based distribution of *Megathyrsus maximus* accessions using phenotypic and genotypic data. **A** K-means clustering based on phenotypic data using the most representative variables from PCA (Dim1 and Dim2); colors indicate cluster membership. **B** Distribution of accessions along the first two genetic principal components (PC1 and PC2), colored by K-means phenotypic cluster assignment. **C** Distribution of accessions along the first two genetic principal components (PC1 and PC2), colored by ancestry coefficients estimated by sNMF (*K* = 3)

Principal Component Analysis (PCA) of the 19 phenotypic variables revealed that flowering (Var1), PH (Var3-Var6), and ADF (Var11) contributed most strongly to the first two principal components (Dim-1 and Dim-2; Supplementary Fig. 2). K-means clustering of the PCA scores identified three distinct phenotypic groups (Fig. 4A). However, these phenotypic clusters showed no clear correspondence with the genetic structure inferred from the sequencing data (Figs. 4B-C). Similarly, PCA based on genetic data showed no strong evidence of population structure among the *M. maximus* accessions (Fig. 4; Supplementary Fig. 3). To account for potential population stratification, the first three principal components were included in the GWAS analysis.

### Selection of a reference genome for single-nucleotide polymorphism calling and genome-wide association study

**Fig. 5:**
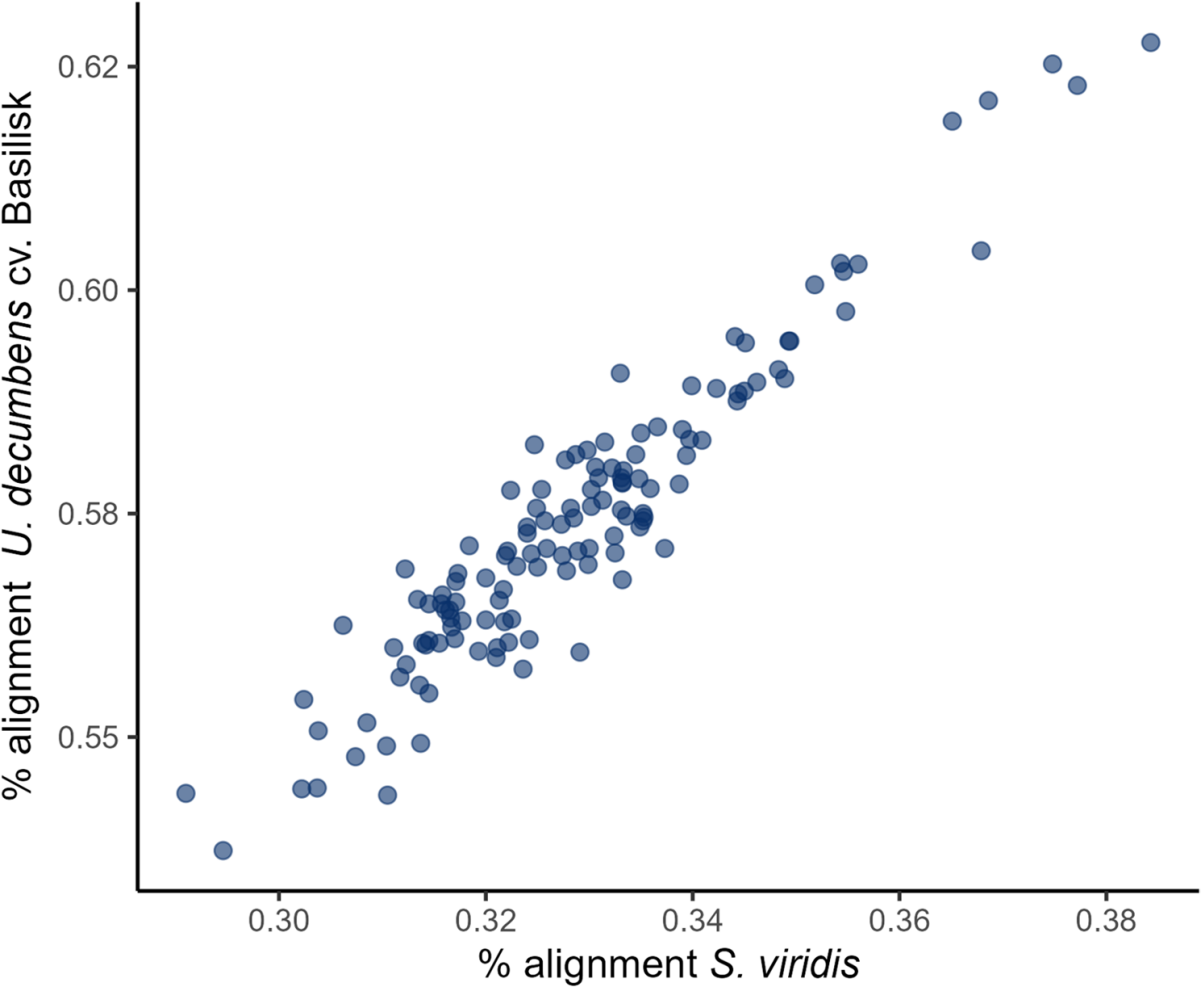
Alignment rates of *Megathyrsus maximus* sequencing reads mapped to *Urochloa decumbens* and *Setaria viridis* reference genomes

In the absence of a reference genome for *M. maximus*, sequencing reads were aligned to the genomes of *Urochloa decumbens* cv. Basilisk [28] and *Setaria viridis* v1.1 [29]. Comparison of alignment rates showed that the *U. decumbens* cv. genome achieved the highest proportion of aligned reads (Fig. 5). Consequently, this genome was selected as the reference for SNP calling and subsequent GWAS analyses.

Linkage disequilibrium (LD) decay analysis showed that LD declined sharply with physical distance, reaching half of its maximum r² value at approximately 146 kb across the *M. maximus* genome (Supplementary Fig. 4). This indicates that genomic segments within ~ 150 kb remain in moderate to strong LD.

### Genome-wide association study and functional annotations

Genome-wide association analyses (GWAS) using three complementary statistical models (BLINK, FarmCPU, and MLMM) across the 19 phenotypic variables identified a total of 722 significant marker-trait associations (MTAs): 216 with BLINK, 250 with FarmCPU and 256 with MLMM (Supplementary Table 2; Supplementary Figs. 5–6). After evaluating all MTAs, we prioritized 25 loci that were detected by at least two models or exhibited a well-defined association peak (a cluster of high-significance SNPs), criteria shown to reduce model-specific biases and false positives. These robust MTAs were distributed across 13 chromosomes (Table 3 and Supplementary Figs. 5–6). Given the polyploid nature of *M. maximus*, the lack of a species-specific reference genome, and the moderate panel size, we applied a genome-wide significance threshold of − log₁₀(p) > 5, a value commonly used in GWAS of tropical forages and polyploid grasses where strict Bonferroni correction is overly conservative [45]. These MTAs covered 11 traits, grouped into: (i) Morphological and phenological traits (flowering, PH); (ii) Nutritional quality traits (CP, IVDMD, ADF, and NDF); and (iii) Biomass production and N-use traits (N uptake, NR, SBP, GFW, and DMY).

**Table 3:** Significant markers identified through GWAS for 11 agronomic and nutritional traits in a diverse panel of *Megathyrsus Maximus* accessions (* markers identified by multiple variables)

Trait	Var	MTA ID	Ref/Alt	*U. decumbens* RefGen			Gene transcription	Variant annotation	Model	p value	-Log10	PVE
				Position	Chr	Scaffold						
Flowering	Var1	1C_56701065	T/C	56,701,065	1 C	9	Udec.scaffold_9G7455.1	Upstream transcript	MLMM	1.84E-06	5.7	1.88
		4D_10705692	T/A	10,705,692	4D	6	Udec.scaffold_6G933.1	Missense	FarmCPU	4.49E-07	6.3	1.48
	Var2	2B_4591364	A/G	4,591,364	2B	7	Udec.scaffold_7G781.1	Upstream transcript	MLMM	2.69E-06	5.5	0.45
									FarmCPU	5.99E-08	7.2	0.45
Plant	Var3	2B_77961460	G/C	77,961,460	2B	7	Udec.scaffold_7G6978.1	Missense	FarmCPU	9.33E-06	5	6.41
	Var4	3B_16691782	G/A	16,691,782	3B	5	Udec.scaffold_5G1138.1	Synonymous	MLMM	1.02E-06	5.9	1.32
									FarmCPU	9.84E-11	10	10.5
	Var5	2B_30705654	G/A	30,705,654	2B	7		Intergenic	MLMM	4.40E-06	5.3	0.62
		6C_58560236	C/T	58,560,236	6 C	3		Intergenic	FarmCPU	4.65E-07	6.3	5.14
									BLINK	7.83E-06	5.1	0.39
	Var6	7C_45623907	T/C	45,623,907	7 C	26	Udec.scaffold_26G2666.1	Upstream transcript	MLMM	1.66E-07	6.7	6.81
									FarmCPU	8.01E-14	13.1	17.2
									BLINK	4.42E-11	10.3	25.6
		7A_53275832	C/T	53,275,832	7 A	24	Udec.scaffold_24G3366.1	5 prime UTR	MLMM	4.56E-07	6.3	1.18
Crude protein (CP)	Var7	5B_53821437	T/A	53,821,437	5B	22	Udec.scaffold_22G5453.1	Splice region	FarmCPU	2.38E-06	5.6	0
									BLINK	1.97E-06	5.7	0
									MLMM	6.21E-06	5.2	0.8
		5B_53821445	A/G	53,821,445	5B	22	Udec.scaffold_22G5453.1	Splice donor & intron	BLINK	2.82E-06	5.5	2.81
									FarmCPU	3.39E-06	5.4	2.81
	Var8	6C_51478739	G/C	51,478,739	6 C	3	Udec.scaffold_3G4207.1	Missense	MLMM	1.13E-06	5.9	0
									FarmCPU	3.41E-06	5.4	0.95
									BLINK	2.84E-06	5.5	0.95
		8A_50124855*	G/A	50,124,855	8 A	19		Intergenic	MLMM	2.29E-06	5.6	5.73
									FarmCPU	5.19E-06	5.2	6.12
									BLINK	4.32E-06	5.3	6.12
In vitro dry matter digestibility (IVDMD)	Var9	5B_2619044	A/T	2,619,044	5B	22	Udec.scaffold_22G388.1	Upstream transcript	BLINK	3.37E-06	5.47	1.79
									FarmCPU	4.05E-06	5.39	1.79
	Var10	8A_50124855*	G/A	50,124,855	8 A	19		Intergenic	FarmCPU	5.41E-06	5.27	1.07
									BLINK	4.42E-06	5.35	1.07
									MLMM	2.40E-06	5.62	4.35
Acid detergent fiber (ADF)	Var11	3B_82457007	G/A	82,457,007	3B	5	Udec.scaffold_5G4191.1	Upstream transcript	BLINK	7.88E-06	5.1	2.78
									MLMM	8.35E-06	5.08	0.52
									FarmCPU	9.33E-06	5.03	2.78
Neutral detergent fiber (NDF)	Var12	6C_63511111	A/G	63,511,111	6 C	3	Udec.scaffold_3G5994.1	Synonymous	MLMM	1.83E-07	6.74	4.18
									BLINK	1.41E-06	5.85	0.13
		3B_76604911	A/G	76,604,911	3B	5		Intergenic	MLMM	2.41E-07	6.62	1.21
									BLINK	8.24E-07	6.08	0.33
									FarmCPU	1.01E-06	5.99	0.33
N uptake	Var13	1C_63236281*	C/T	63,236,281	1 C	9	Udec.scaffold_9G8098.1	Missense	BLINK	3.14E-06	5.5	1.72
		9B_45223860	C/T	45,223,860	9B	32	Udec.scaffold_32G2943.1	Intron	FarmCPU	5.50E-06	5.26	3.17
									MLMM	5.37E-06	5.27	
Nitrification Rate	Var14	4D_45206878	TA/T	45,206,878	4D	6	Udec.scaffold_6G2682.1	Splice region	FarmCPU	1.47E-06	5.83	13.6
									BLINK	5.15E-06	5.29	0
									MLMM	2.83E-07	6.55	0
		9A_14592912	C/T	14,592,912	9 A	34	Udec.scaffold_34G1571.1	Intron	FarmCPU	2.55E-08	7.59	3.66
									BLINK	3.98E-13	12.4	0.06
Shoot biomass production (SBP)	Var15	1C_63236281*	C/T	63,236,281	1 C	9	Udec.scaffold_9G8098.1	Missense	BLINK	5.20E-06	5.28	1.78
	Var16	5D_16245287	C/T	16,245,287	5D	16	Udec.scaffold_16G1981.1	Intron variant	MLMM	2.46E-06	5.61	0.79
									BLINK	2.56E-07	6.59	1.53
									FarmCPU	3.19E-07	6.5	1.53
Dry Matter Yield (DMY)	Var17	2C_8e_97872092	C/T	97,872,092	2C_8e	4	Udec.scaffold_4G8704.1	Synonymous	BLINK	9.81E-06	5.01	0.66
	Var18	5D_16245273	A/T	16,245,273	5D	16	Udec.scaffold_16G1981.1	Intron	BLINK	3.41E-06	5.47	4.87
									FarmCPU	4.08E-06	5.39	4.87
	Var19	6C_57335884	T/A	57,335,884	6 C	3	Udec.scaffold_3G5110.1	Intron	FarmCPU	3.95E-06	5.4	0.36
									BLINK	1.06E-08	7.98	0
									MLMM	3.03E-07	6.52	0

**Fig. 6:**
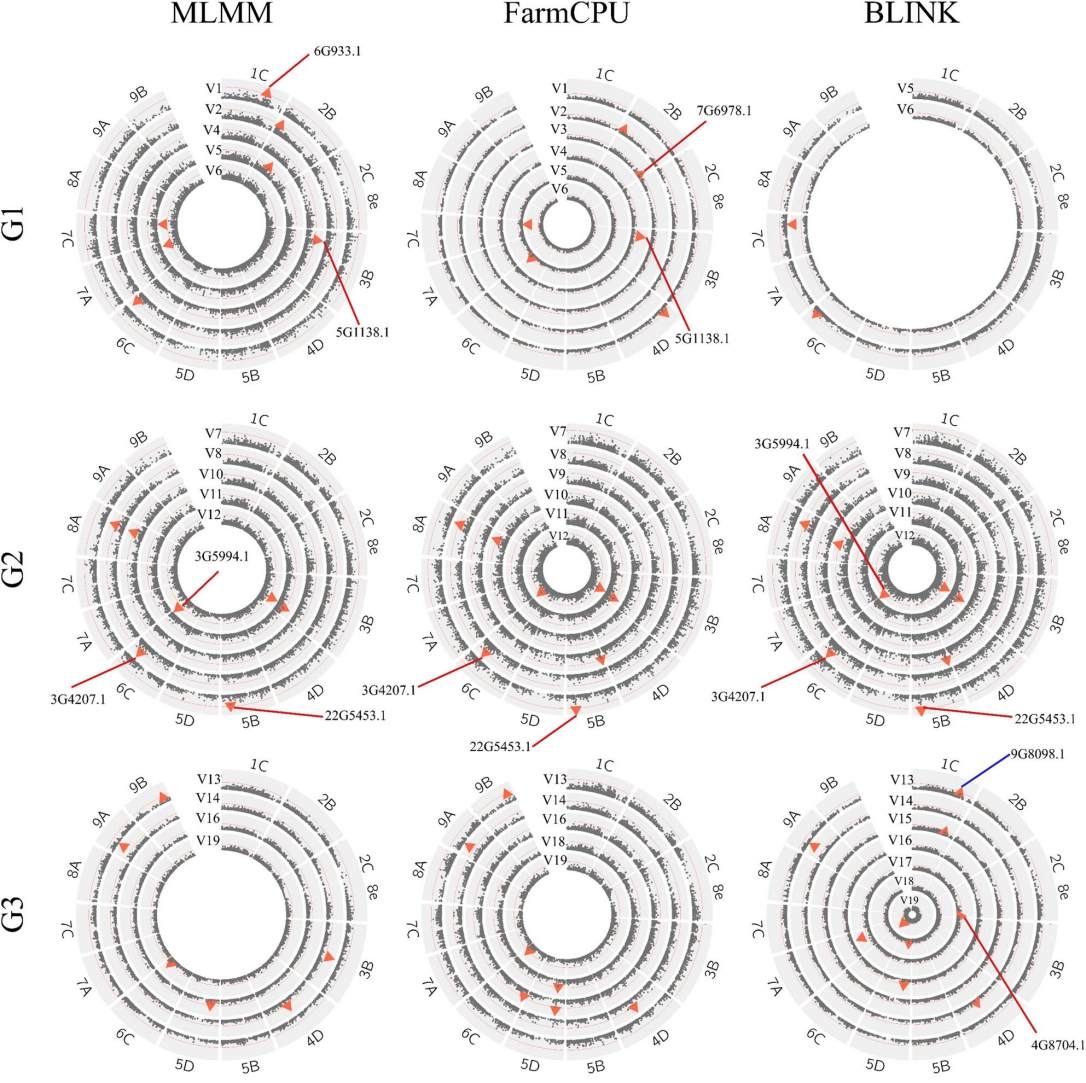
Genome-wide Association Study (GWAS) results across trait categories for *M. maximus*. Circular Manhattan plots summarizing the 25 significant marker–trait associations (MTAs) detected across the 19 phenotypic variables (V1-V19), grouped into three major trait categories: (G1) Morphological and phenological traits (flowering, PH); (G2) Nutritional quality traits (CP, IVDMD, ADF, and NDF); and (G3) Biomass production and nitrogen-use traits (N uptake, NR, SBP, GFW, and DMY). Results from the three GWAS models (BLINK, FarmCPU, and MLMM) are displayed, with the red line indicating the significance threshold (− log₁₀(p) = 5). MTAs located within coding regions of candidate genes are annotated to highlight putative functional loci

**Fig. 7:**
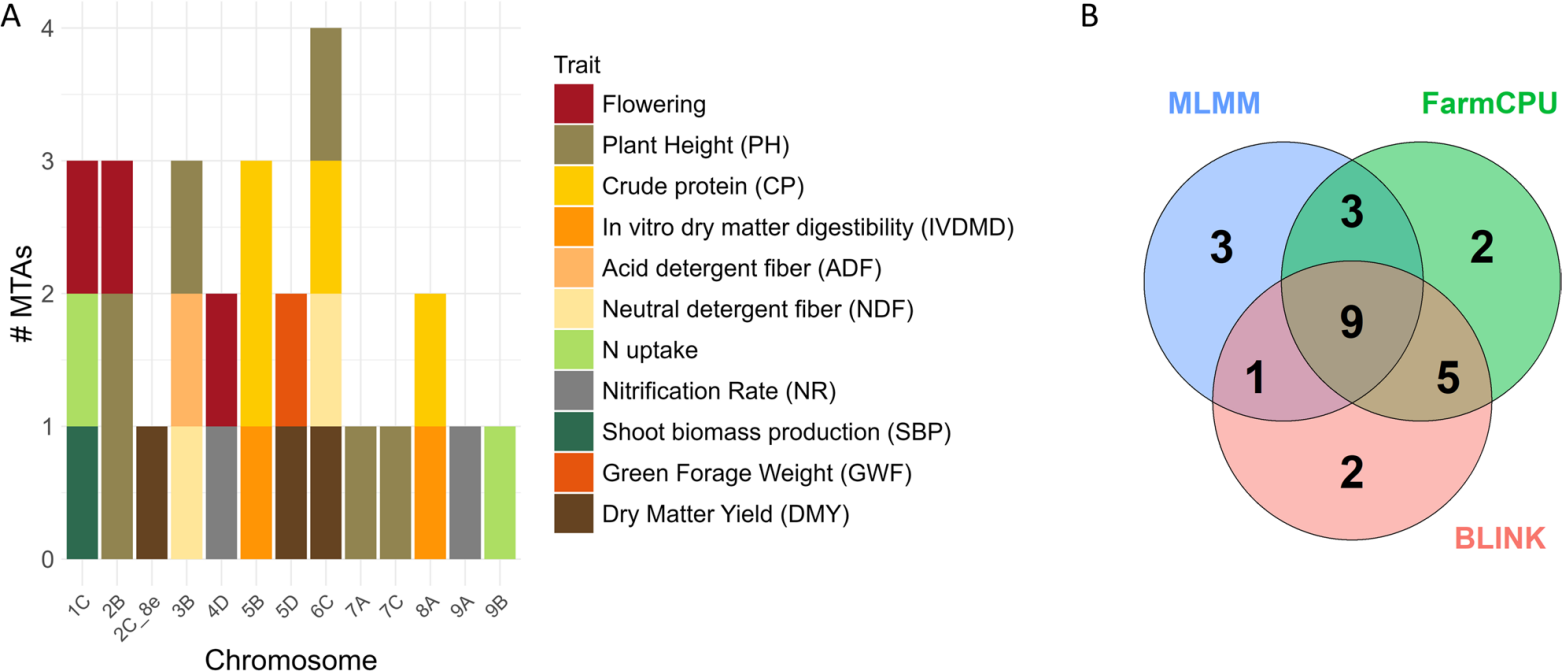
Summary of GWAS results and comparison across models. **A** Chromosomal distribution of the 25 marker–trait associations (MTAs) identified using *U. decumbens* cv. Basilisk reference genome (Ryan et al., 2025). **B** Venn diagram illustrating the overlap and model-specific detection of significant markers among the three GWAS approaches (BLINK, FarmCPU, and MLMM)

Across all models, the proportion of phenotypic variation explained (PVE) by individual MTAs ranged from 0 to 25.58% (mean = 3.14%). BLINK detected 17 MTAs, MLMM identified 16, and FarmCPU detected 19 (Table 3; Figs. 6 and 7). Nine markers were consistently detected by all three models (3B_76604911, 3B_82457007, 4D_45206878, 5D_16245287, 5B_53821437, 6C_51478739, 6C_57335884, 7C_45623907, and 8A_50124855), while two models shared additional markers. Each model also identified between 2 and 3 markers not detected by the other models (Table 3; Fig. 7). QQ plots for all trait–model combinations showed good agreement between observed and expected p-values, indicating that population structure and kinship were adequately controlled (Supplementary Fig. 5).

### Morphological and phenological traits

For flowering percentage (Var1–Var2), three significant MTAs were identified on chromosomes 1 C, 2B, and 4D (Table 3; Figs. 6 and 7). Marker 1C_56701065, linked to flowering during the dry season (Var1), was located within a gene encoding a pentatricopeptide repeat (PPR) protein, a family known to regulate organelle biogenesis and reproductive development. Marker 2B_4591364, associated with wet-season flowering (Var2), was positioned near genes encoding helix–loop–helix transcription factors involved in photoperiodic control of flowering. A second flowering-associated locus during the dry season (Var1), 4D_10705692 on chromosome 4D, was also annotated as a PPR protein. For PH (Var3–Var6), six MTAs were identified across chromosomes 2B, 3B, 6 C, 7 A, and 7 C, including the strongest association detected in the study (Figs. 6 and 7; Table 3). Marker 7C_45623907 was consistently identified by all three GWAS models and explained the highest proportion of phenotypic variance (25.58%). Although the SNP itself was located in a non-protein-coding region, it lies near genes encoding zinc-ribbon and kinase domains known to influence internode elongation. Two markers on chromosome 2B (2B_77961460 and 2B_30705654) were associated with PH during wet-season evaluations (Var3 and Var5, respectively). The first is a missense variant linked to a gene involved in dihydrokaempferol 4-reductase activity, while the second is an intergenic variant located near a gene encoding a peptidase S10 family protein. On chromosome 3B, marker 3B_16691782, detected by both MLMM and FarmCPU, was a synonymous variant situated within a gene encoding an ankyrin-repeat protein (SKIP35). Marker 6C_58560236 on chromosome 6 C, associated with Var5, was positioned near members of the myosin gene family, which are implicated in cellular expansion. Finally, marker 7A_53275832 on chromosome 7 A (Var6) accounted for 1.18% of the phenotypic variance.

### Nutritional quality traits

For CP (Var7–Var8) and IVDMD (Var9–Var10), five MTAs were identified across chromosomes 5B, 6 C, and 8 A. For CP, marker 6C_51478739 (Var8) was annotated as a missense variant within a gene encoding an ATP-binding cassette (ABC) transporter C family member. Two additional CP-associated markers on chromosome 5B (5B_53821437 and 5B_53821445, Var7) mapped to a genomic region enriched for genes involved in transcriptional regulation and cytoskeletal organization. The first was annotated as a splice-region variant within a gene encoding the C-terminal domain of RNA polymerase Rpb5, whereas the second was located near genes encoding actin-binding proteins, tubulin, and RNA polymerase sub**units (Supplementary Table 3). Fo**r IVDMD, marker 5B_2619044 (Chr 5B, Var9) was located within a gene essential for siRNA biogenesis in the RNA-silencing pathway, with a nearby gene encoding a coatomer complex component implicated in intracellular protein transport. The second association, 8A_50124855 (Chr 8 A, Var10), was shared with CP (Var8), suggesting a potential genetic link between protein content and digestibility (Table 3). Although this SNP lies outside annotated protein-coding regions, it resides within a gene of unknown function and warrants further investigation.

The remaining nutritional quality traits, ADF and NDF, yielded three significant MTAs across chromosomes 3B and 6 C, all of which were consistently detected by multiple GWAS models (Table 3; Figs. 6 and 7). For ADF (Var11), marker 3B_82457007 (Chr 3B) was identified as an upstream transcript variant near a gene encoding an adenosine diphosphate (ADP)-binding protein. Two markers were associated with NDF (Var12): 3B_76604911 (Chr 3B), located outside protein-coding regions, and 6C_63511111 (Chr 6 C), positioned within a gene encoding a nonaspanin family protein, also known as transmembrane 9 (TM9) superfamily proteins (TM9SF, TC 9.A.2) (Table 3; Supplementary Table 3). These associations point to genomic regions influencing fiber biosynthesis and cell-wall composition, key determinants of forage digestibility and nutritional value.

### Biomass production and N-use traits

For N uptake (Var13), two MTAs were identified on chromosomes 1 C and 9B (Table 3; Figs. 6 and 7). Marker 1C_63236281 was associated with both N uptake and SBP (Var15), and was annotated as a missense variant within a gene of unknown function, located near transcription factors of the bHLH-MYC and R2R3-MYB families, regulators known to influence growth and metabolic pathways (Supplementary Table 3). This marker explained 1.72% of the phenotypic variance. The second association, 9B_45223860, was an intronic variant within a gene encoding the catalytic sub-unit of ferredoxin–thioredoxin reductase (FTR), a key enzyme in redox regulation, explaining 3.17% of the variance. For NR (Var14), two significant MTAs were detected (Table 3; Figs. 6 and 7). Marker, 4D_45206878, accounted for 13.64% of the phenotypic variance and corresponded to a splice-region variant in a gene encoding a Rhodanese Homology Domain protein, potentially affecting enzyme function relevant to nitrogen cycling. The second association, 9A_14592912, was an intronic variant located near genes encoding ankyrin-repeat and GAG-pre-integrase domains, suggesting possible regulatory functions related to soil–plant nitrogen interactions.

Shoot biomass production (SBP, Var15), GFW (Var16), and DMY (Var17–Var19) also showed five significant genomic associations (Table 3; Figs. 6 and 7). As noted above, the marker 1C_63236281 on chromosome 1 C was shared between N uptake and SBP, highlighting a genomic region potentially influencing both N-use efficiency and biomass accumulation. On chromosome 5D, two closely positioned markers (5D_16245287 and 5D_16245273) were associated with GFW and DMY, respectively. The first was detected by all three GWAS models and mapped near genes encoding F-box proteins, ribosomal protein L5, and subtilase family members (Supplementary Table 3). Marker 5D_16245273 was annotated as an intron variant, located within genes encoding the 50 S ribosomal protein L5 and subtilases, and suggesting potential regulatory roles in growth and tissue development. An association with DMY was also observed on chromosome 6 C at 6C_57335884, consistently detected across all three models; nearby genes included those encoding phosphoinositide kinases and AP2-domain transcription factors. An additional MTA, 2C_8e_97872092, was detected on chromosome 2C_8e for DMY, further highlighting the polygenic nature of yield-related traits in *M. maximus*. Together, these associations point to multiple genomic regions controlling biomass production.

Collectively, these 25 MTAs provide genomic insights across three major trait categories and highlight candidate genes with potential relevance for improving forage quality, biomass production, and N-use efficiency in *M. maximus*. In summary, from the total MTAs identified, 40% (10 variants) were located within coding regions, including missense (*n* = 4), synonymous (*n* = 3), and splice-region variants (*n* = 3), all of which may directly affect gene function or transcript processing. The remaining 60% (15 variants) were classified as non-coding, comprising intronic (*n* = 5), upstream transcript (*n* = 5), intergenic (*n* = 4), and 5′ UTR variants (*n* = 1). These non-coding variants may influence regulatory elements, transcriptional activity, or gene expression rather than protein structure. Together, the diversity of variant types reflects a broad spectrum of potential functional mechanisms underlying trait variation in *M. maximus*, spanning both protein-coding changes and regulatory modulation.

## Discussion

Characterizing genebank forage collections and mining genomic regions associated with key traits provides valuable opportunities for future breeding programs [14, 16, 46]. In our study, the analysis of 124 *M. maximus* accessions from the CIAT forage collection revealed three distinct genetic clusters, as identified through WGS using sNMF and hierarchical clustering methods. However, no clear genetic structure was observed with respect to country of origin. Different factors may explain this pattern: (i) extensive movement or dispersion of germplasm across regions; (ii) the outcrossing reproductive biology of some sexual *M. maximus* populations, which promotes genetic mixing and reduces geographic differentiation; (iii) convergent selection pressures in environmentally similar locations, leading to genetic and phenotypic similarities independent of geographic distance; and (iv) imprecise or incomplete passport data for some accessions, which can obscure true geographic patterns. Together, these factors help explain the minimal clustering by geographical origin observed in our results. Comparable clustering results have been reported in other studies: Villegas et al. [13], focusing on BNI, identified three phenotypic clusters among 119 accessions, including high-BNI group advanced/improved cultivars such as Tobiata (CIAT6299) and Mombasa (CIAT6962) [13]. Likewise, Carvajal-Tapia et al. [21] reported three clusters based on nutritional quality traits. Despite differences in methodology, these studies consistently highlight the existence of three primary groups within *M. maximus*. This convergence has practical breeding implications, as identifying genetic groups enriched for superior nutritional and BNI-related traits can guide targeted improvement. Building on the demonstrated success of *Urochloa* breeding programs that exploited BNI traits to reduce soil nitrification [47], similar strategies could be applied in *M. maximus* to combine strong agronomic performance with ecosystem services, including N_2_O emissions mitigation from soil.

Few genome-trait association studies have been conducted across grass forages, with examples including *Cenchrus ciliaris*, *Cenchrus purpureus*, and *Dactylis glomerata* [16, 48, 49]. To date, no such analyses have been reported for *M. maximus*, largely due to the absence of a publicly available reference genome. In this study, we addressed this gap by performing GWAS using a diverse panel of genebank accessions and evaluating 11 key traits. Although several phenotypic variables deviated from normality, the GWAS methods used in this study (BLINK, FarmCPU, and MLMM) are designed to be robust to non-normal trait distributions. Therefore, these deviations are not expected to substantially compromise the reliability of the marker–trait associations detected. Sequencing reads were aligned to the *Urochloa decumbens* cv. Basilisk, as a reference genome, a related species selected after comparison with another potential reference genome based on mapping efficiency. Twenty-five significant MTAs were identified across 13 chromosomes for traits previously reported in *M. maximus* [13, 21]. These included flowering time, PH, CP content, IVDMD, ADF, NDF, N uptake, NR, SBP, GWF, and DMY. These traits are critical determinants of forage quality, productivity, and nutritional value, and directly influence both livestock performance and environmental impact.

Flowering, a trait strongly correlated with PH, N uptake, and SBP [21], showed significant genetic associations across chromosomes 1 C, 2B, and 4D. Two MTAs were located near genes encoding: (i) F-box protein involved in the jasmonate signaling pathway, which regulates early flowering [15, 50]; and (ii) helix–loop–helix domain proteins, known for their role in photoperiodic control and flowering time regulation in rice [51]. A third MTA, identified on chromosome 4D, was annotated as a missense variant within a gene encoding a pentatricopeptide repeat (PPR) protein. PPR proteins are essential for organelle biogenesis, pollen development, and overall plant growth in rice (for example) [52]. Within a 40 kb window surrounding this marker, two non-synonymous mutations were also detected, potentially influencing flowering [43, 44]. However, none of the flowering MTAs were consistently detected across all models. Identifying genes underlying flowering regulation is pivotal for optimizing reproductive timing in forage breeding programs. These genetic insights provide a foundation for understanding molecular mechanisms of flowering and can guide breeding strategies to increase seed yield potential.

Plant height (PH) is a key trait in forage breeding due to its high heritability and strong positive correlation with DMY [21, 53]. In our study, PH also showed strong correlations with DMY and flowering time. Six MTAs linked to this trait were detected across chromosomes 2B, 3B, 6 C, 7 A, and 7 C, annotated as either coding or non-coding variants. Of these, only one, 7C_45623907, was consistently detected across all three models, exhibiting the highest PVE, ranging from 6.81% to 25.58%. This marker, annotated as an upstream transcript variant, was located near a gene encoding a probable zinc-ribbon domain and protein-kinase domain, both of which regulate PH and internode elongation in maize (*Zea mays*) [54]. Among the remaining MTAs, one on chromosome 2B was annotated as a missense variant detected by FarmCPU, explaining 6.41% of the PVE. This variant encodes a protein with dihydrokaempferol 4-reductase activity, a key enzyme in the flavonoid biosynthetic pathway in anthocyanin production and freezing stress response in *Brassica rapa* [55]. It also plays a role in auxin regulation, influencing plant growth and flowering time in *Nicotiana tabacum* [56].

Crude protein (CP) content in forages reflects the available protein, which is necessary to meet livestock requirements for growth, reproduction, and milk production [3]. In this study, two CP variables, measured in the dry (Var7) and wet (Var8) seasons, were associated with loci on chromosomes 5B, 6 C, and 8 A. Three of these four associations were consistently detected across the MLMM, FarmCPU, and BLINK models. Notably, one wet-season association on chromosome 6 C was annotated as a missense variant within a gene encoding an ATP-binding cassette (ABC) transporter C family member. This locus was located near genes encoding disease resistance proteins (NB-ARC, RPP13-like, pectinesterase inhibitor), and included non-synonymous and frameshift variants [43, 57]. During the wet season, seasonal effects on CP content were evident. Rapid plant growth contributes to higher CP levels in younger tissues. However, during the dry season, plant maturity shifts resources towards structural components such as fibers, reducing CP content. These dynamics highlight the importance of candidate MTAs for CP content in managing forage quality and ensuring balanced livestock nutrition year-round.

Moreover, IVDMD—an indicator of overall forage quality—exhibited two associations on chromosomes 5B and 8 A. The latter (8A_50124855) was consistently detected by all three models as an intergenic variant, with a PVE value ranging from 1.07% to 4.35%. Interestingly, this locus was also associated with CP content. Functional annotation identified nearby genes of unknown function, but the region harbored four high-impact non-synonymous mutations, suggesting potential functional effects that warrant further investigation [43, 44].

Fiber composition traits, including ADF and NDF, are key indicators of forage quality and digestibility. Higher ADF and NDF values reflect greater fiber content, which is typically associated with reduced digestibility, whereas lower values are linked to improved nutritional value for livestock [3, 58]. In our study, NDF showed a moderate negative correlation with plant height (-0.71), while ADF displayed a moderate positive correlation with flowering (0.71). These patterns suggest that taller plants may allocate more resources to structural components such as lignin and cellulose, the main constituents of ADF and NDF, which can decrease forage quality if fiber levels become excessive.

The GWAS identified three MTAs for fiber composition traits: one for ADF and two for NDF. Each trait included one association consistently detected across all three models, annotated as upstream transcript and intergenic variants on chromosome 3B (3B_82457007) and 6 C (6C_63511111), respectively. These loci were located within genes encoding an ADP-binding protein and a member of the transferase family. Transferases such as 3-O-methyltransferase (COMT) are known to reduce lignin content in alfalfa (*Medicago sativa*) [59], while phosphatidylinositol 4-kinase (PIK4), another transferase, has been implicated in the trafficking of cellulose synthase complexes [60]. Consistently, Lin et al. [14] reported a marker in *M. sativa* (MtChr8_30029709) associated with NDF, also located within the PIK4 gene region.

Nitrogen (N) uptake and NR are crucial traits in forage systems because of their direct impact on N utilization and metabolism. Higher N uptake enhances protein production, thereby improving forage nutritional value for livestock [61]. Nitrification rate (NR), defined as the microbial conversion of ammonium (NH4^+^) to nitrate (NO3^−^), is equally important because nitrate is the primary form of nitrogen absorbed by plants [62]. However, excessive nitrification can result in nitrate leaching and groundwater contamination [63]. In our study, N uptake showed a strong positive correlation with SBP and a negative correlation with flowering, while NR was only weakly correlated with the other traits. The GWAS identified four MTAs for N uptake and NR, with one MTA consistently detected by all three models with a PVE of up to 13.64%. This locus, 4D_45206878, associated with NR, was annotated as a splice-region variant within a gene encoding a Rhodanese Homology Domain. Another SNP within the same gene causes a start codon loss, potentially altering gene function. Within the surrounding 40 kb LD block, a gene encoding the ubiquinol–cytochrome c reductase complex 6.7 kDa subunit was detected. This protein is involved in redox reactions and ATP synthesis, and its role in Complex III may influence ammonia oxidation in *Nitrosomonas europaea*, an ammonia-oxidizing bacterium [64]. Importantly, forages such as *M. maximus* are known to exhibit BNI, a plant-driven mechanism that suppresses ammonia oxidation by targeting enzymes like ammonia mono-oxygenase (AMO) [13, 65]. The association identified here may therefore be linked to BNI-related traits and should be further investigated for breeding programs, targeting varieties with enhanced BNI capacity. Incorporating BNI into breeding pipelines could position *M. maximus* as a dual-purpose tropical forage: delivering high yields and quality feed while mitigating N_2_O emissions. Future work should validate these MTAs in larger populations and assess their stability under field conditions to ensure their reliable deployment in breeding programs.

Shoot biomass production (SBP), GFW, and DMY are pivotal traits for forage productivity due to their direct influence on biomass accumulation and feed supply. We identified five MTAs associated with these traits. For SBP, one association (1C_63236281) was detected on chromosome 1 C and also linked to N uptake, suggesting a role in N use efficiency. This SNP was annotated as a missense variant within a gene of unknown function but located near transcription factors of the bHLH-MYC and R2R3-MYB families. These regulators control flavonoid and lignin biosynthesis, key pathways in N uptake and plant growth [66, 67]. In rice, bHLH genes such as LAX1 also influence plant architecture by regulating branching and tillering, with implications for crop productivity [68]. Green forage weight (GFW) and DMY were highly correlated (0.99). Two MTAs (5D_16245287 and 5D_16245273), located only 14 kb apart, were identified within this region. Candidate genes included those encoding the 50 S ribosomal protein L5 and members of the subtilase family. In wheat, *TaL5* expression is induced under stress conditions and is associated with starch accumulation during grain filling [69] while subtilases in rice have been implicated in grain yield and caryopsis quality [70]. Overall, the PVE of the identified MTAs was modest. Among the 25 MTAs detected, only three (two associated with PH and one with NR) explained more than 10% of the phenotypic variance. This relatively low explanatory power may reflect issues of genetic integrity in the seed lots: the material used for DNA extraction and sequencing may not have been identical to the lots used for generating phenotypic data in previous years. Potential errors in tracking or handling of seed lots could have introduced noise, reducing both the significance and the PVE of the associations. In addition, because an annotated reference genome for this species is not yet available, SNP calling was performed using the genome of a related species. While this was a necessary choice to enable association analysis, it may have introduced biases in SNP detection and reduced the accuracy of effect-size estimates, further contributing to the modest PVE values observed. On the other hand, although LD decay extended to ~ 146 kb, we adopted a more conservative 40 kb window for candidate-gene searches around MTAs. This window size remains well below the LD decay threshold and restricts the analysis to genomic regions most likely to contain physically linked causal variants, thereby minimizing the inclusion of unrelated genes. Similar window sizes (20–50 kb) have been used in GWAS of other cross-pollinated tropical forage grasses with comparable LD structure, supporting the suitability of this approach [71, 72]. Therefore, while the MTAs identified in this study represent a valuable foundation for trait-targeted breeding in *M. maximus*, further functional validation is needed to confirm the causal genes underlying these associations. Future work using model species such as *Arabidopsis* or rice, or through genome-editing approaches in tropical forages, will be essential to experimentally validate candidate genes and strengthen the biological interpretation of GWAS signals.

## Conclusions

This study targeted key traits of tropical forage breeding, aiming to identify genomic regions essential for developing high-yielding varieties with improved nutritional quality and environmental co-benefits. By leveraging a closely related forage grass as a reference genome, we adopted an innovative strategy to detect trait-associated regions, resulting in the identification of multiple MTAs. To our knowledge, this is the first GWAS conducted in *M. maximus* using a diverse panel of genebank accessions, including wild materials and three advanced cultivars, without known pedigree relationships, providing valuable markers for accession selection and the potential to reduce evaluation time in breeding programs. The *M. maximus* cv Mombasa reference genome is currently under assembly and development [73] representing an important step toward a more comprehensive genomic characterization of this important forage species, and providing a valuable foundation for understanding the genetic architecture of target traits. Importantly, the integration of phenotypic data collection under both wet and dry conditions from previous studies strengthened the robustness of our findings, providing insights into species adaptation and resilience to contrasting environments. The use of three complementary statistical models minimized false positives and reinforced the reliability of the genomic regions identified. The MTAs identified in this study provide the foundation for downstream gene discovery, marker-assisted selection, and the development of genomic selection panels.

Overall, our results underscore the central role of GWAS in improving tropical forage breeding to advance livestock productivity and deliver environmental benefits, such as reduced N losses. This study highlights the current state of genomic resources and statistical methods available for *M. maximus*, outlining their potential to accelerate tropical forage improvement. By linking genomic associations to traits such as biomass, BNI, digestibility, flowering, and protein content, this research establishes a foundation for breeding *M. maximus* cultivars that address the dual challenges of food security and climate-change mitigation. The study represents an important step towards the practical implementation of genomic tools in tropical forage breeding programs.

## Supplementary Information

Supplementary Material 1.

Supplementary Material 2.

## Data Availability

The datasets generated and/or analyzed during the current study are available at the European Nucleotide Archive (ENA) at EMBL-EBI under Project PRJEB97636 ( https://www.ebi.ac.uk/ena/browser/view/PRJEB97636).

## Abbreviations

ABC: ATP-binding cassette
ADF: Acid detergent fiber
BLINK: Bayesian-information and Linkage-disequilibrium Iteratively Incorporating Knowledge
BLUPs: Best Linear Unbiased Predictors
BNI: Biological Nitrification Inhibition
CP: Crude protein
D: Dry
DMY: Dry matter yield
FarmCPU: Fixed and Random Model Circulating Polynomial Unification
G: Greenhouse
GWAS: Genome-Wide Association Study
GFW: Green forage weight
IVDMD: In vitro dry matter digestibility
LD: Linkage disequilibrium
MLMM: Multiple Loci Mixed Model
MTAs: Marker-trait associations
NDF: Neutral detergent fiber
NR: Nitrification rate
PCA: Principal Component Analysis
PH: Plant Height
PIK4: phosphatidylinositol 4-kinase
PPR: Pentatricopeptide repeat
PVE: Phenotypic variation explained
SBP: Shoot biomass production
W: Wet
