# Supplementary material for: Genome-wide association study of a Guinea grass (Megathyrsus maximus) diversity panel reveals the genetic basis of agronomic and nutritional traits
Source: BMC Plant Biol. 2025 Dec 30;25:1785. doi: 10.1186/s12870-025-08007-2 (PMC12754869; doi:10.1186/s12870-025-08007-2)

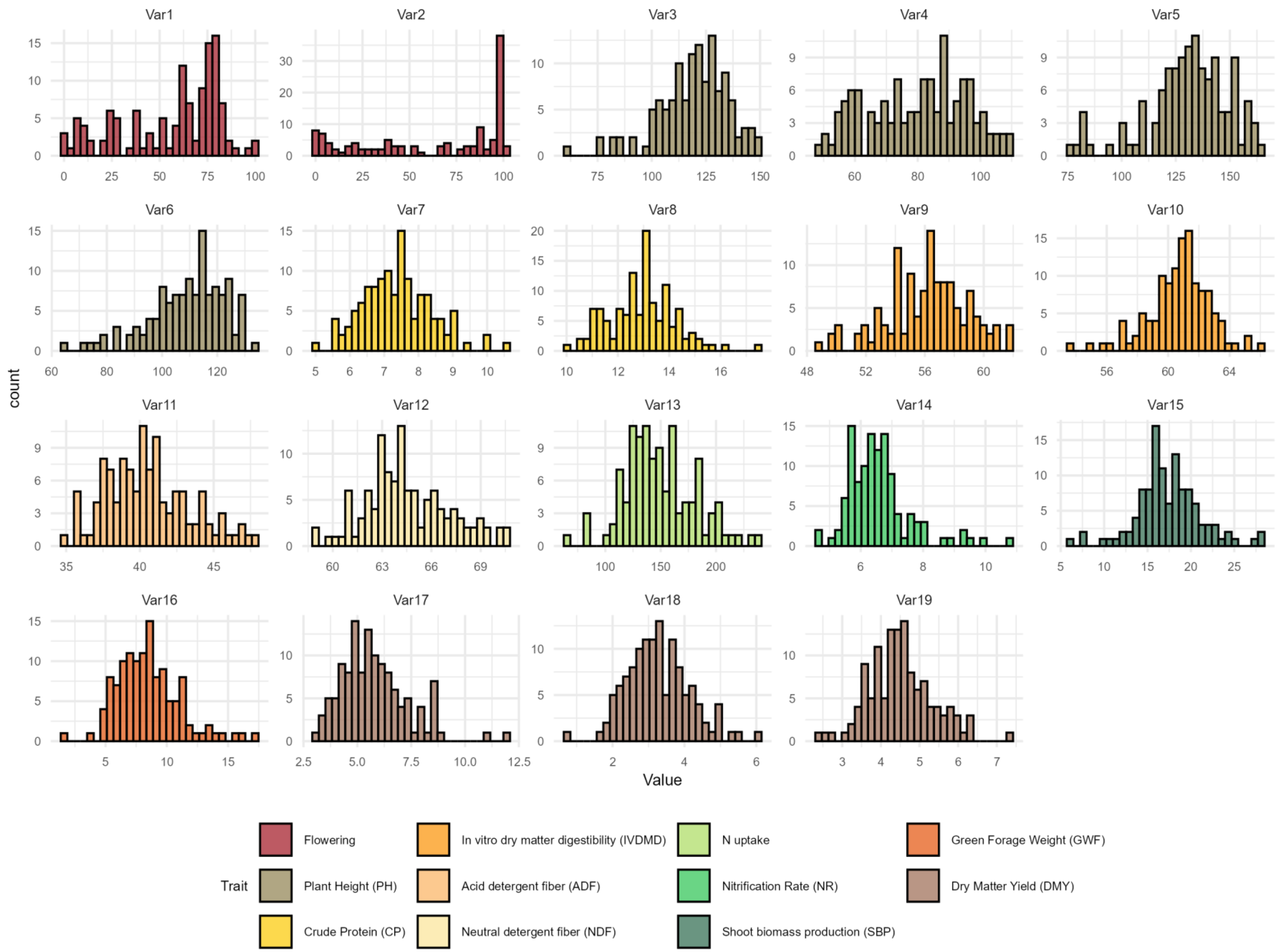

Supplementary Figure 1. Histograms illustrating the distribution of the 19 variables, corresponding to 11 phenotypic traits, analyzed in this study.

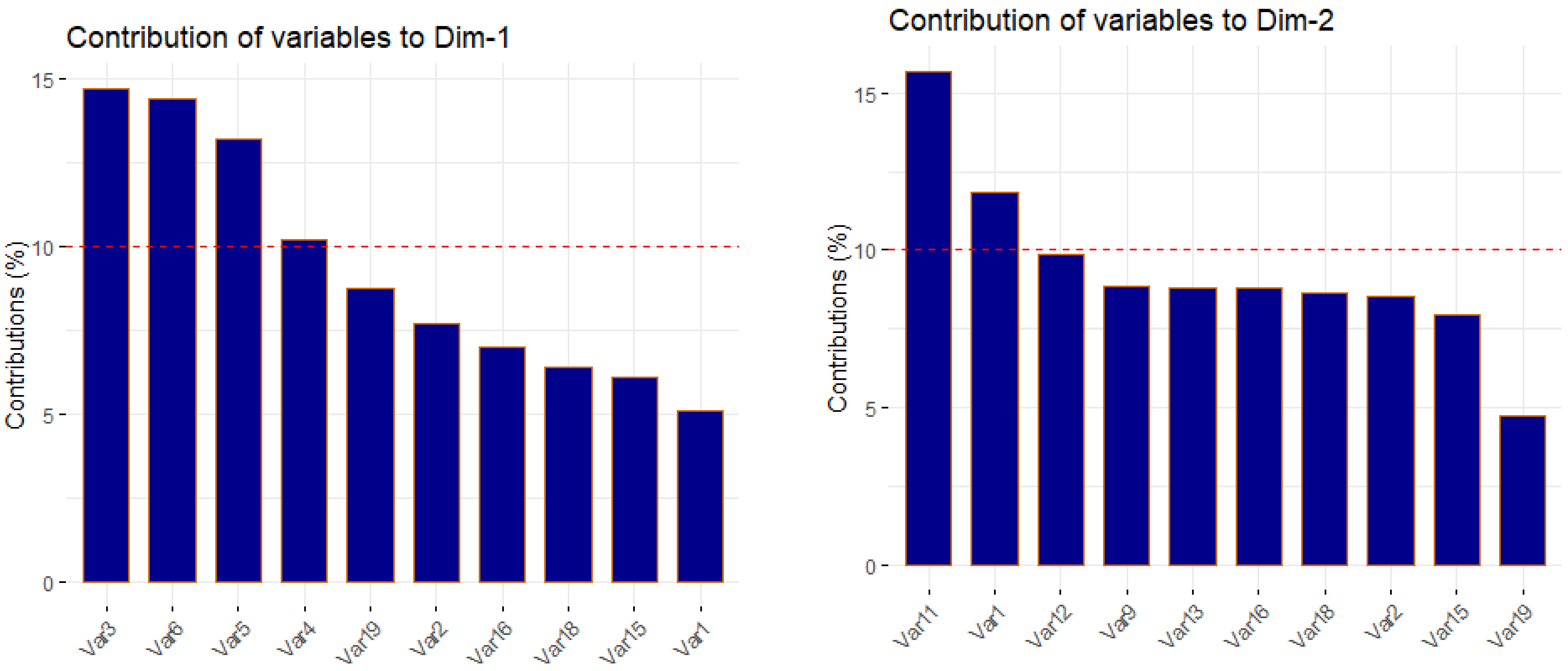

Supplementary Figure 2. Contribution of phenotypic variables to PCA Dimension 1 and Dimension 2.

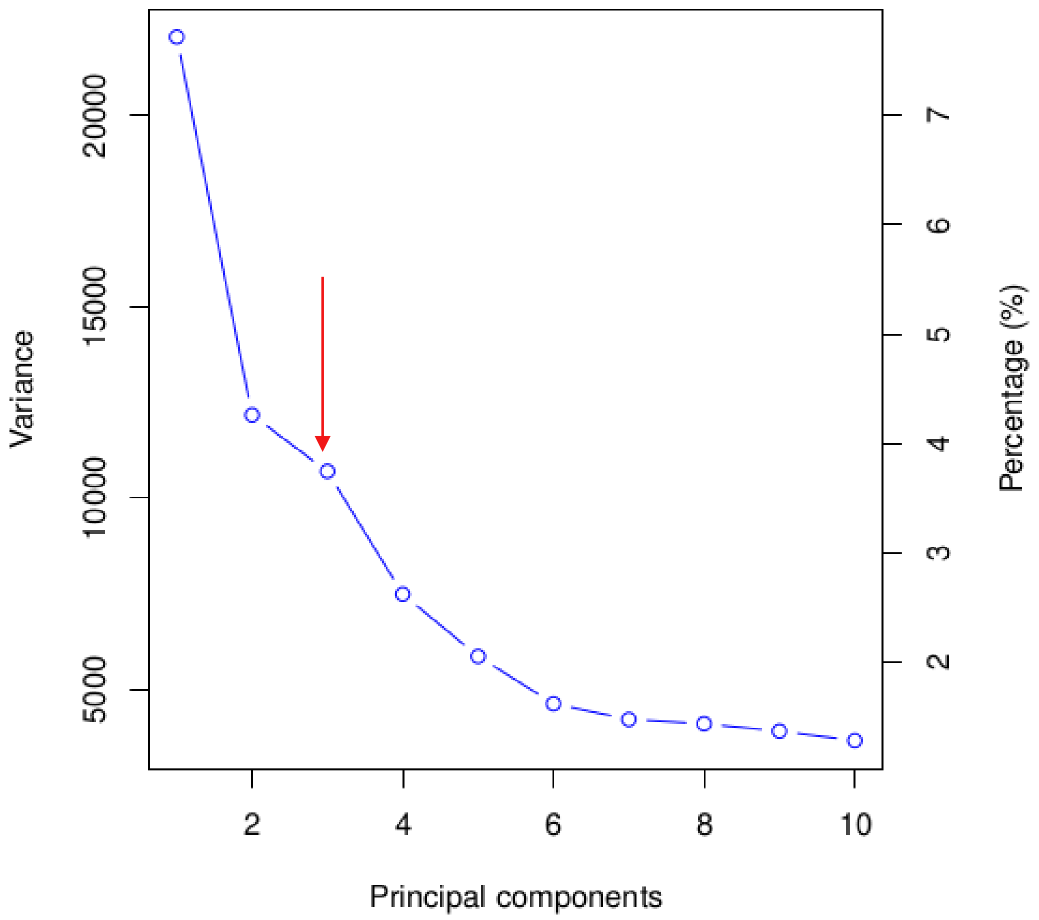

Supplementary Figure 3. Depicts the variance explained by the first ten principal components and their respective percentages, with the red arrow indicating the third principal component (PC3).

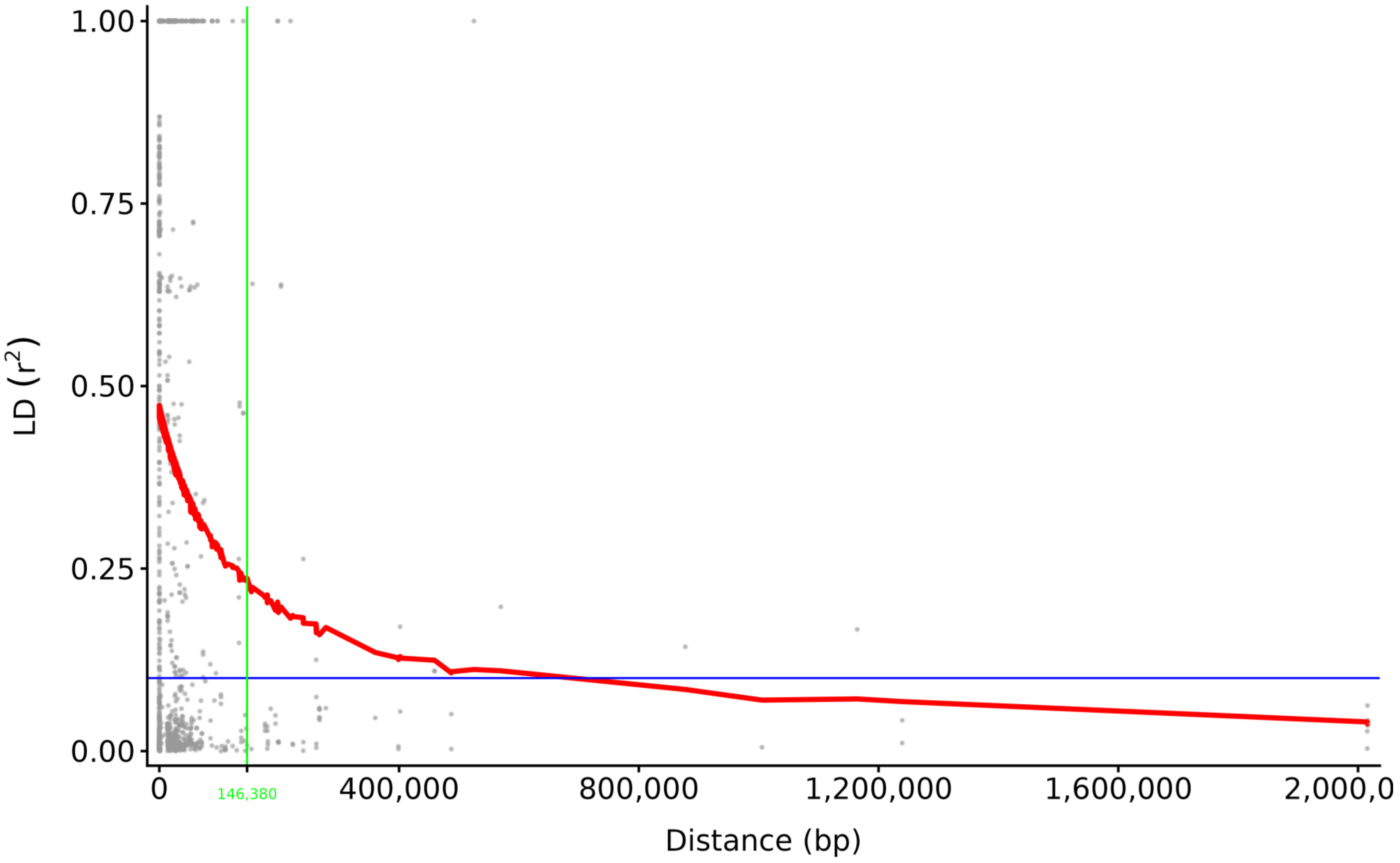

Supplementary Figure 4. Linkage disequilibrium (LD) decay in *Megathyrsus maximus* based on 1,261,156 SNPs across 124 accessions. LD decay ($r^2 = 0.2$) occurred at 146,380 bp using the *Urochloa decumbens* cv. Basilisk reference genome.

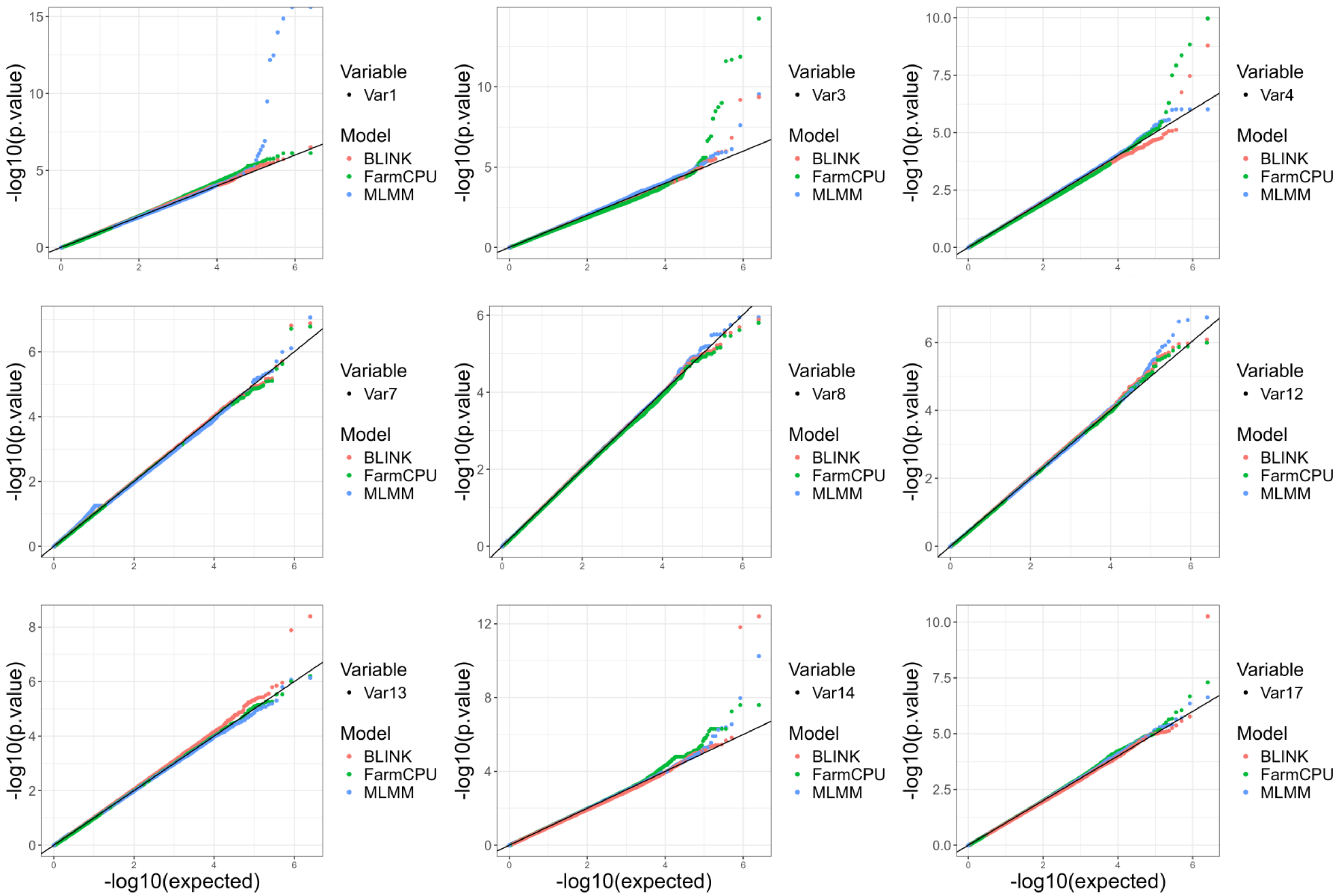

Supplementary Figure 5. Quantile–quantile (QQ) plots of nine marker–trait associations (MTAs) located in protein-coding regions, showing expected and observed SNP test probabilities across the BLINK, FarmCPU, and MLMM GWAS models.

MLMM Flowering (Var 1)

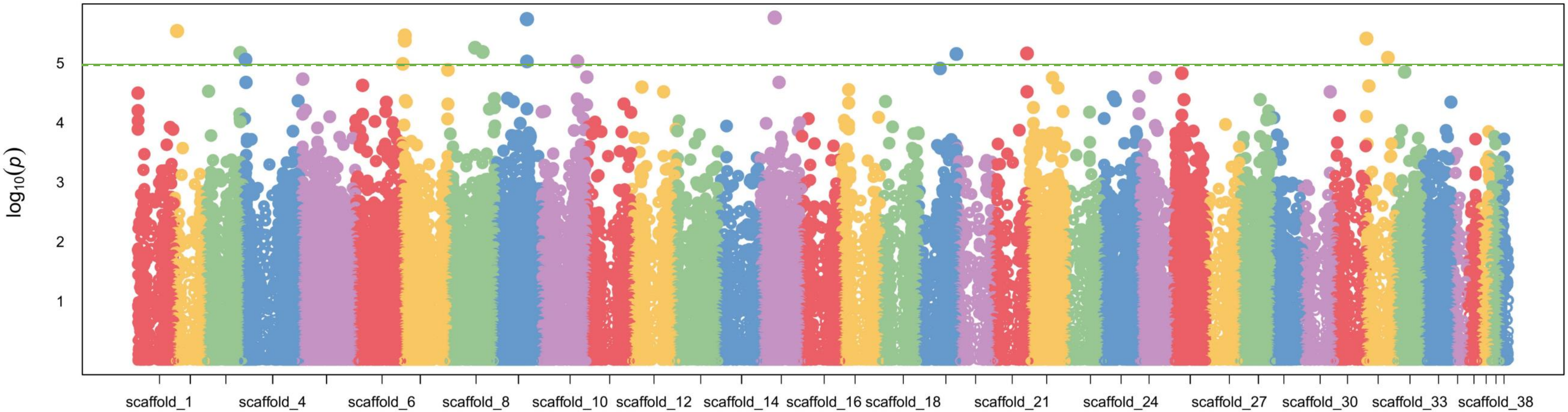

Supplementary Figure 6. Manhattan plots from genome-wide association analyses in *Megathyrsus maximus*

FarmCPU Flowering (Var 1)

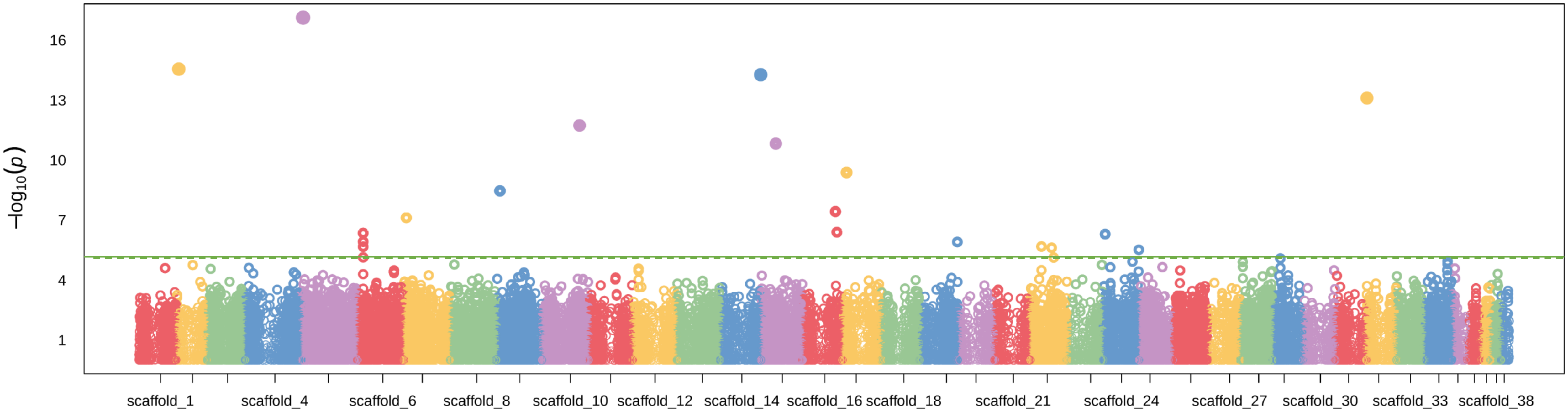

MLMM Flowering (Var 2)

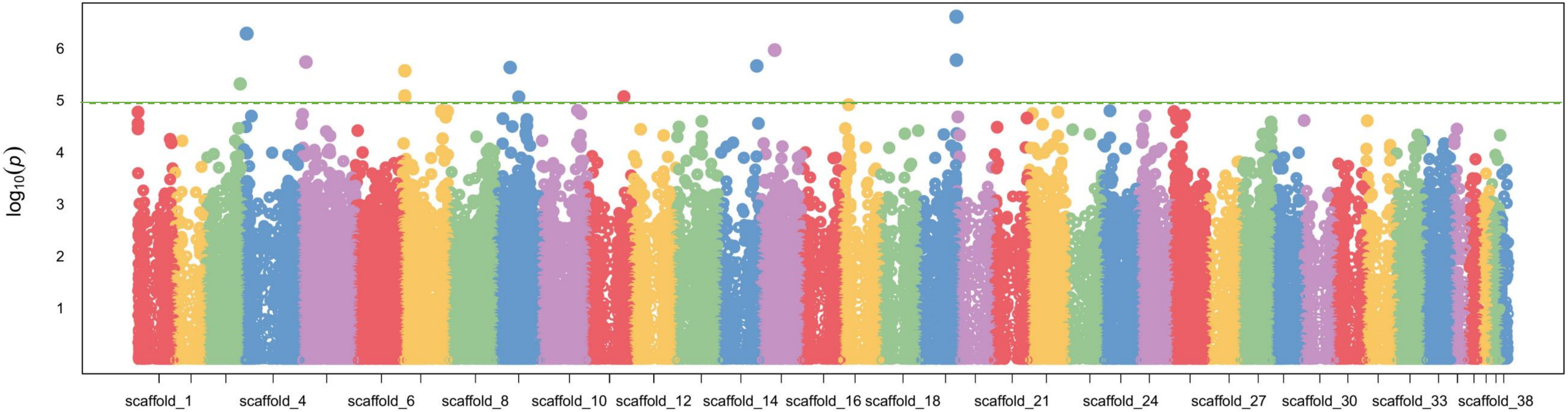

FarmCPU Flowering (Var 2)

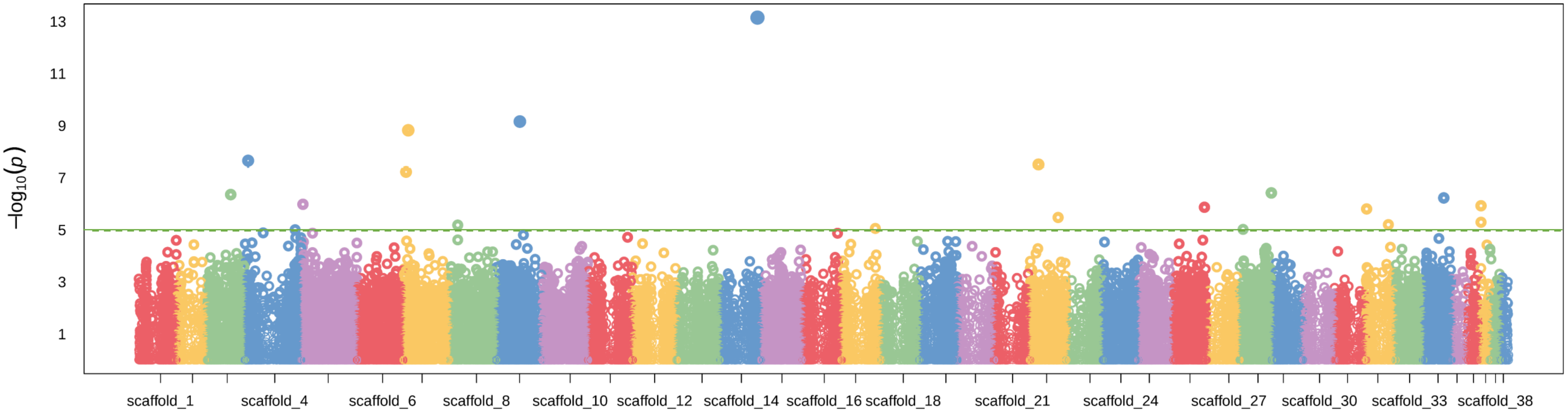

FarmCPU Plant Height (PH) (Var 3)

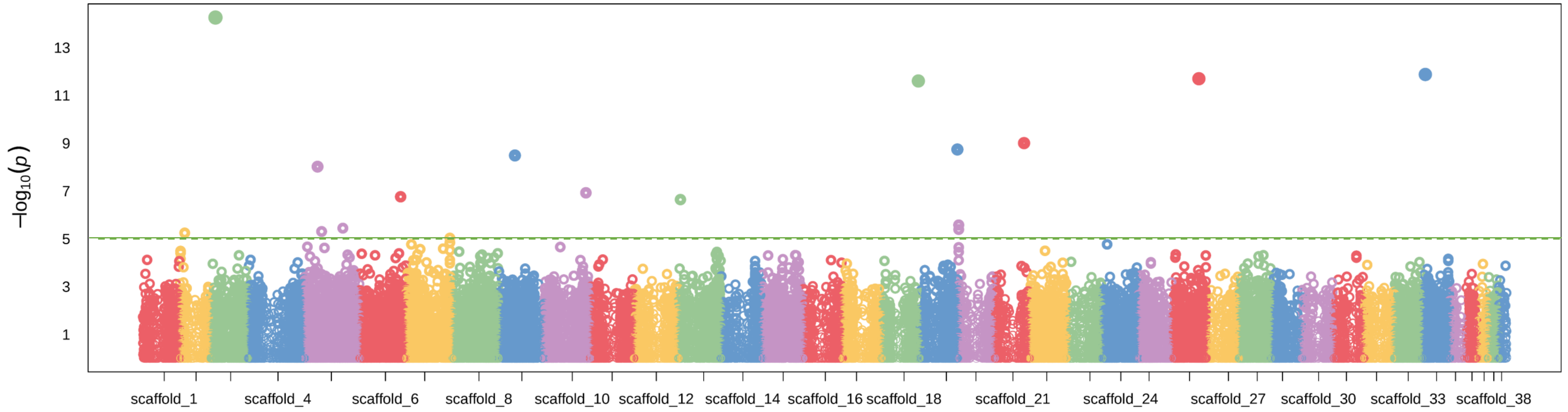

MLMM Plant Height (PH) (Var 4)

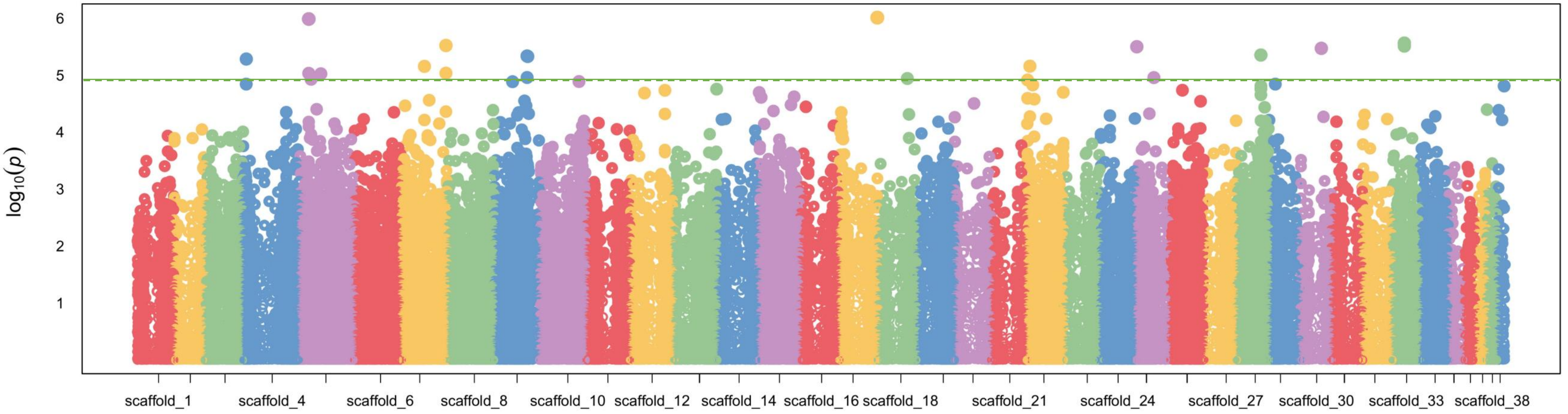

FarmCPU Plant Height (PH) (Var 4)

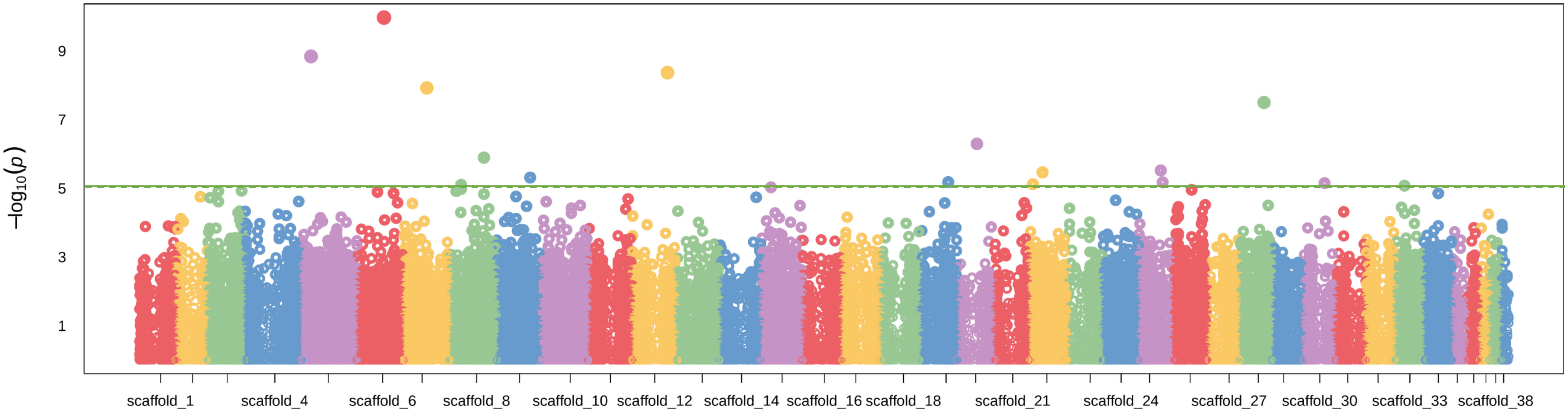

MLMM Plant Height (PH) (Var 5)

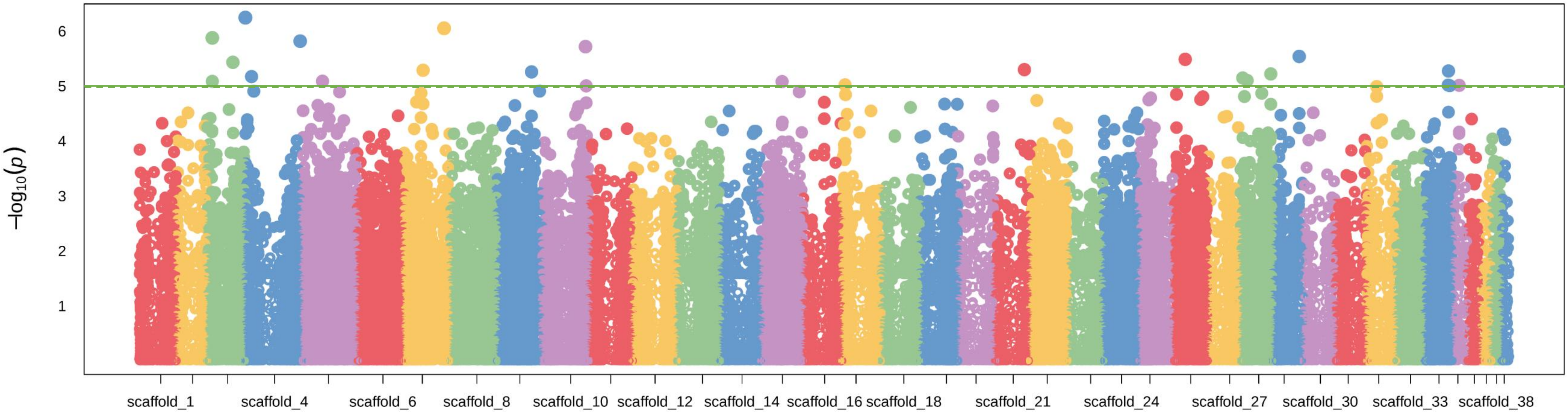

FarmCPU Plant Height (PH) (Var 5)

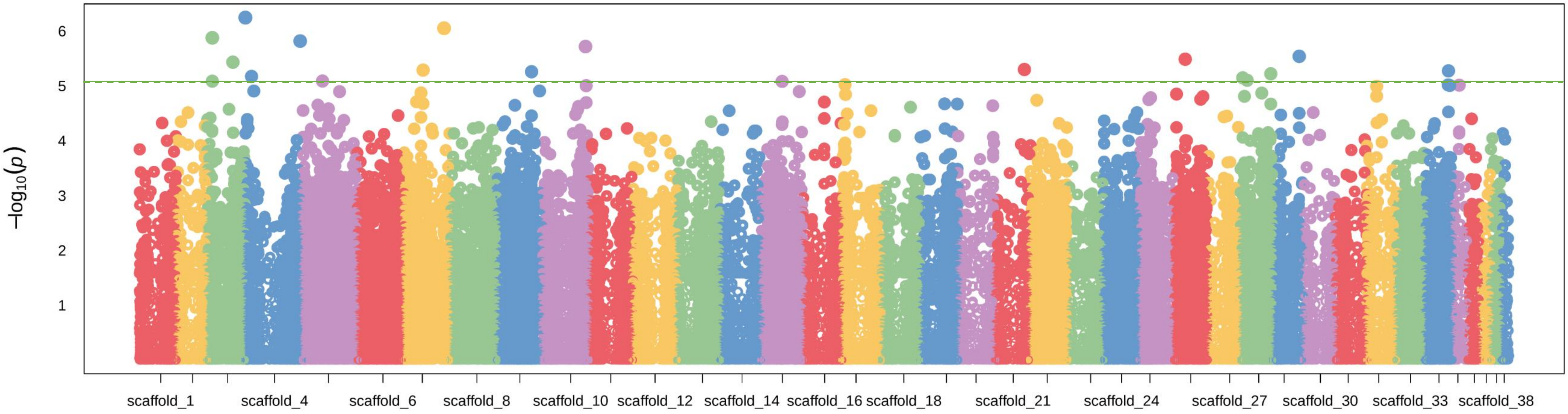

BLINK Plant Height (PH) (Var 5)

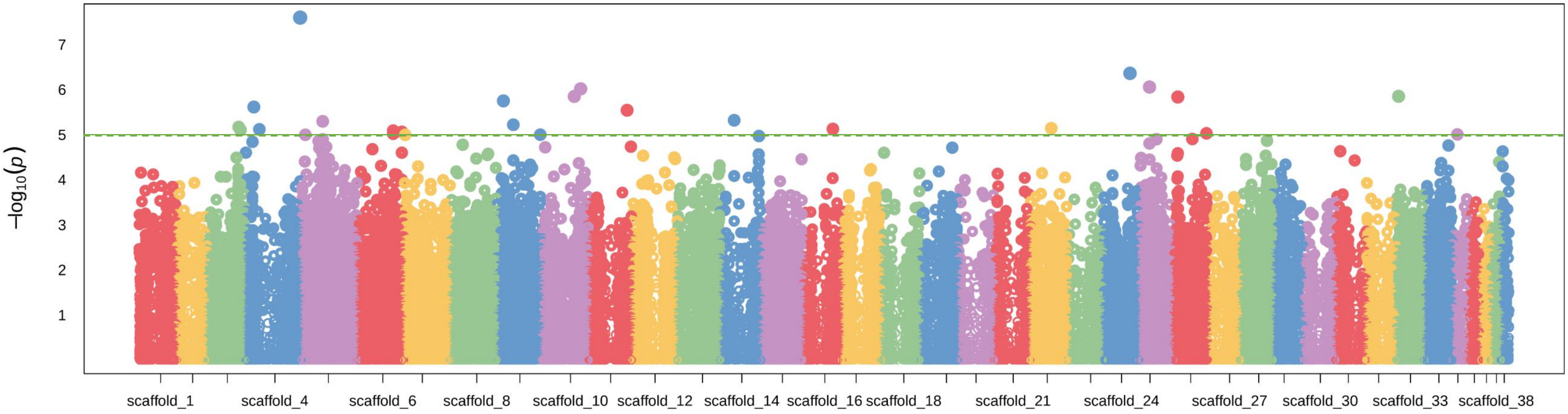

FarmCPU Plant Height (PH) (Var 6)

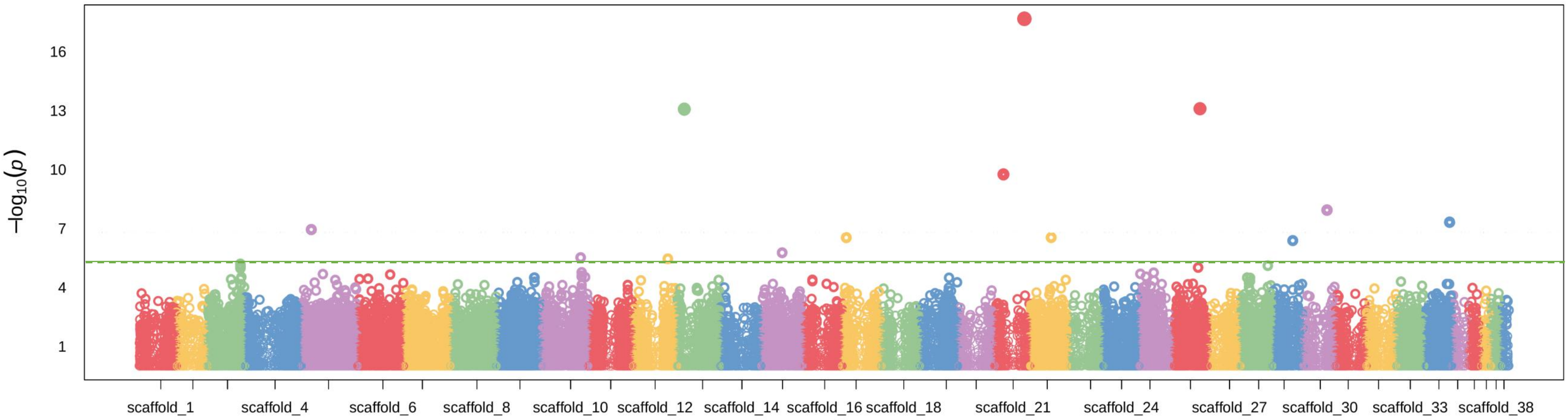

BLINK Plant Height (PH) (Var 6)

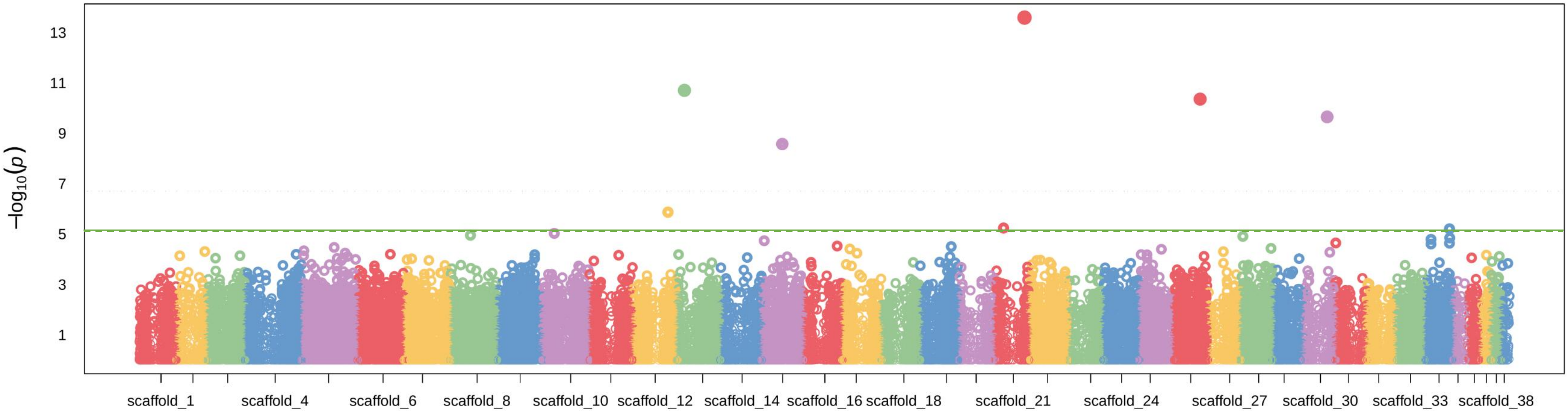

FarmCPU Crude Protein (CP) (Var 7)

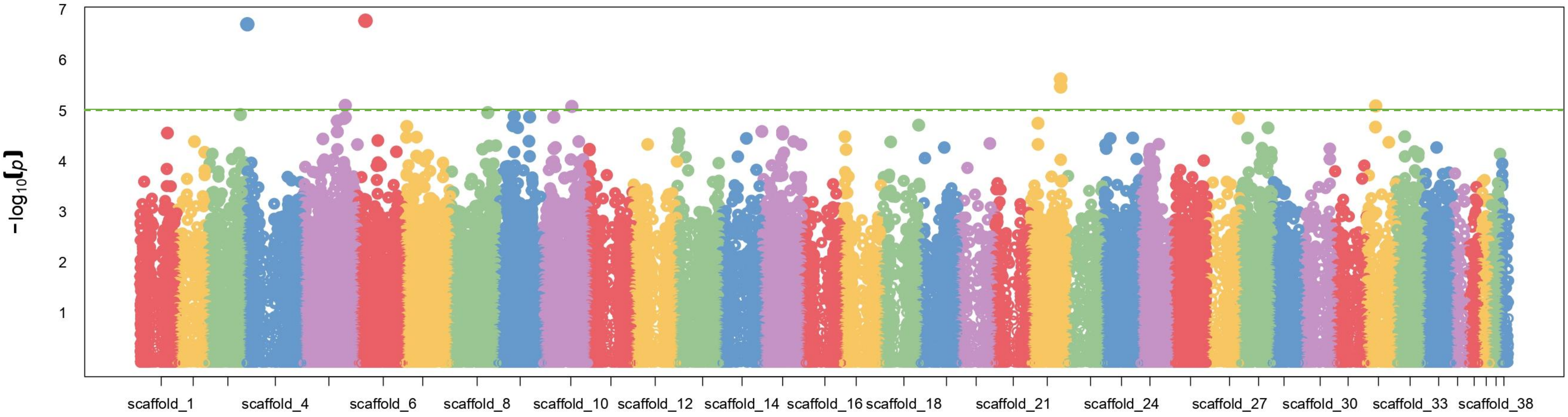

BLINK Crude Protein (CP) (Var 7)

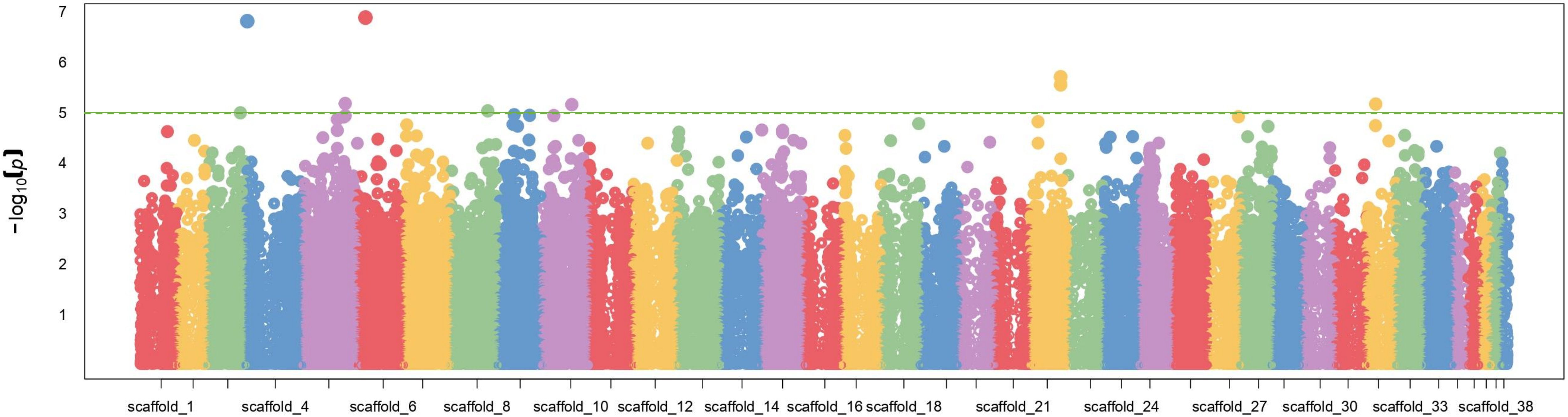

MLMM Crude Protein (CP) (Var 7)

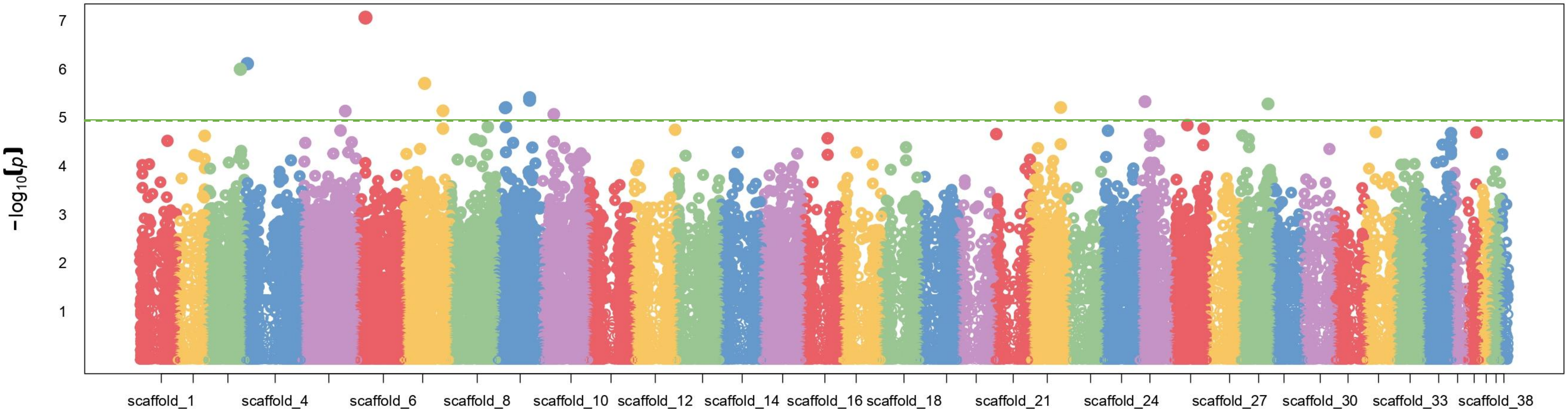

MLMM Crude Protein (CP) (Var 8)

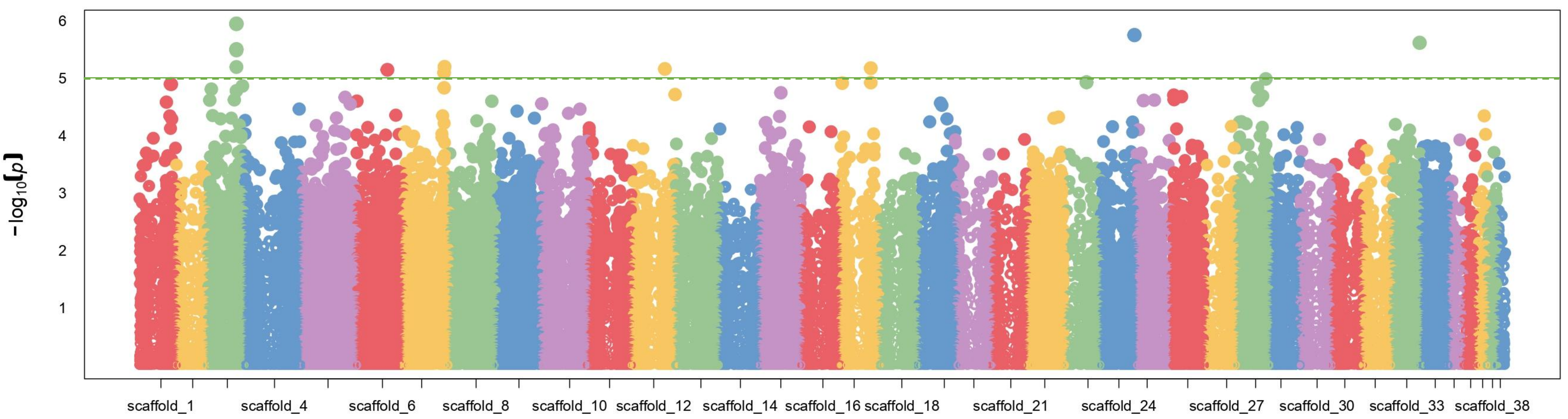

FarmCPU Crude Protein (CP) (Var 8)

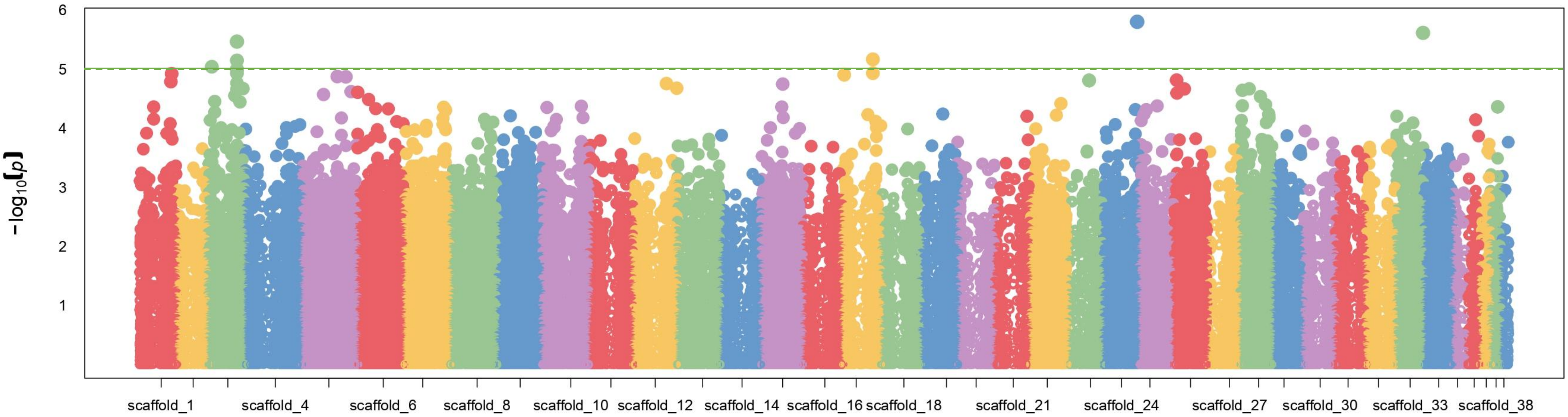

BLINK Crude Protein (CP) (Var 8)

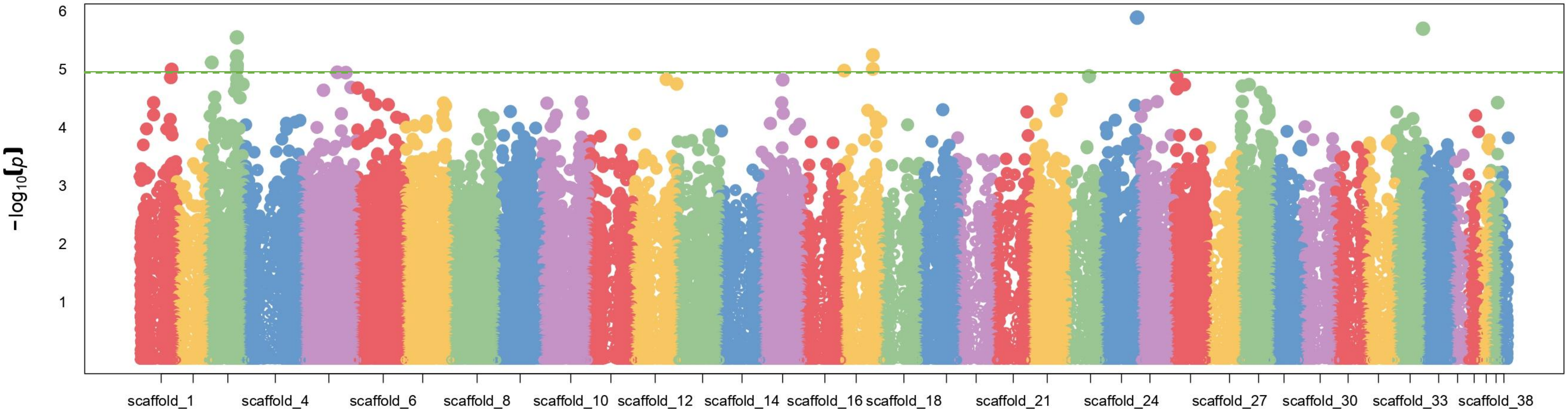

BLINK In vitro dry digestibility (IVDMD) (Var 9)

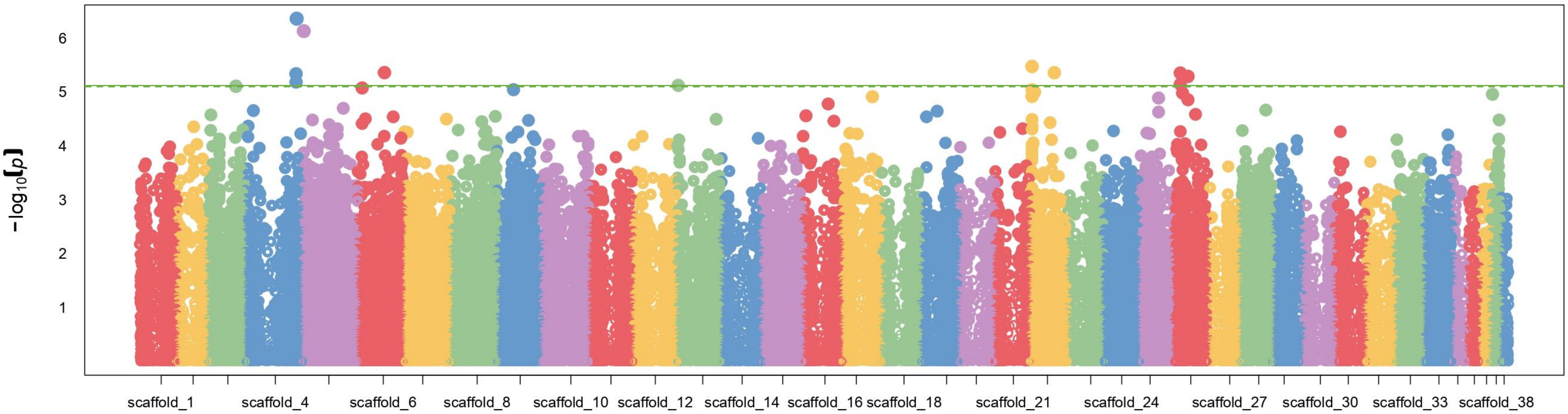

FarmCPU In vitro dry digestibility (IVDMD) (Var 9)

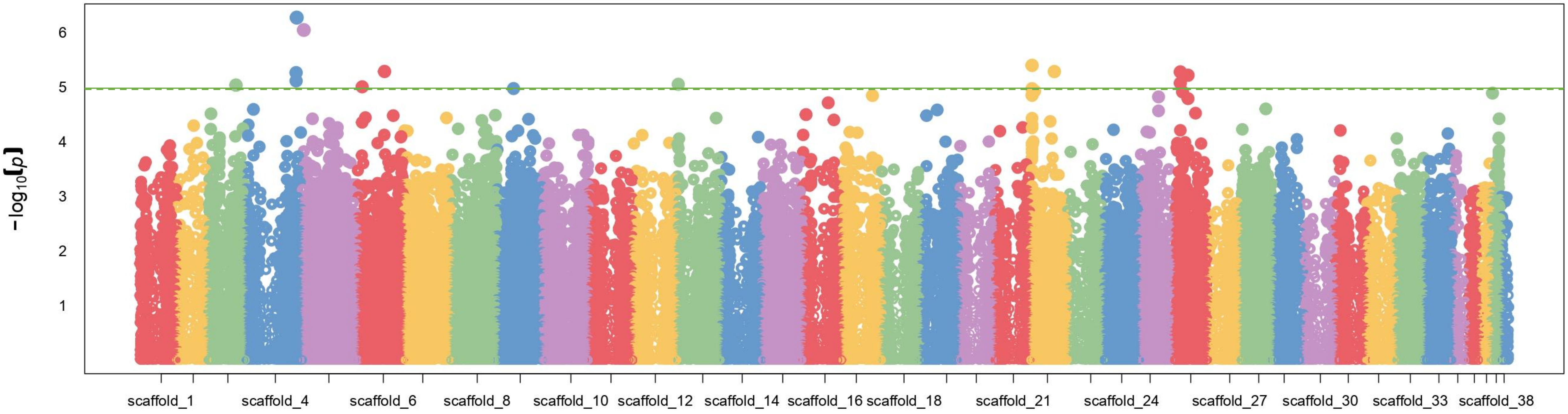

FarmCPU In vitro dry digestibility (IVDMD) (Var 10)

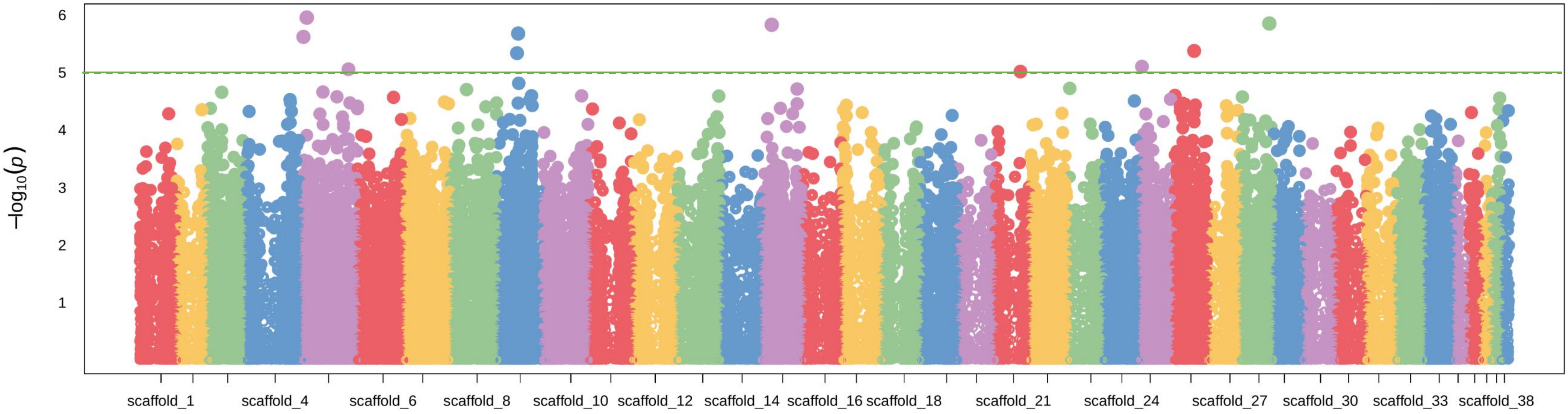

BLINK In vitro dry digestibility (IVDMD) (Var 10)

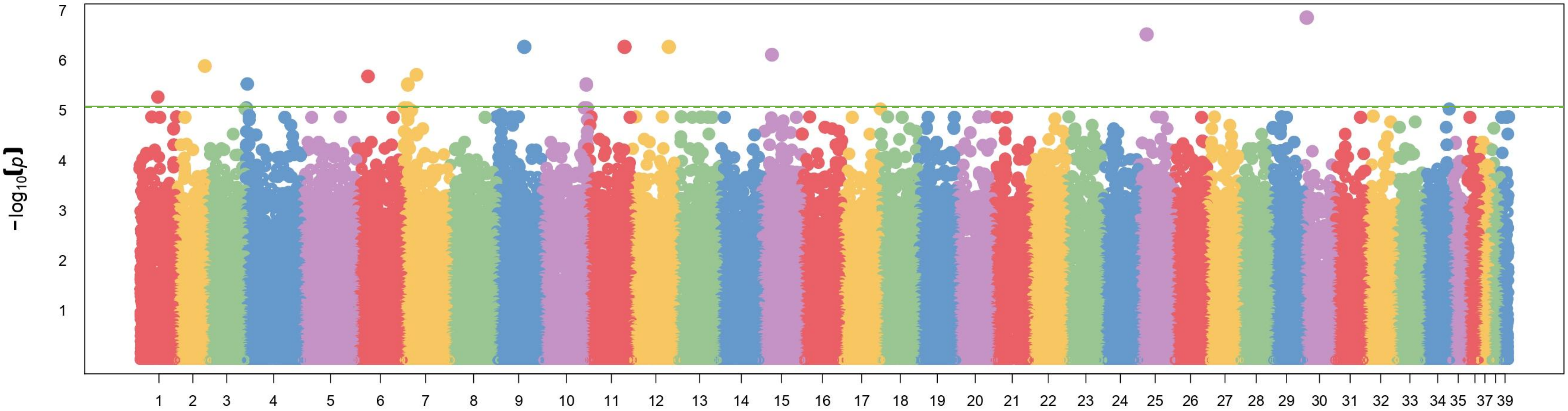

MLMM In vitro dry digestibility (IVDMD) (Var 10)

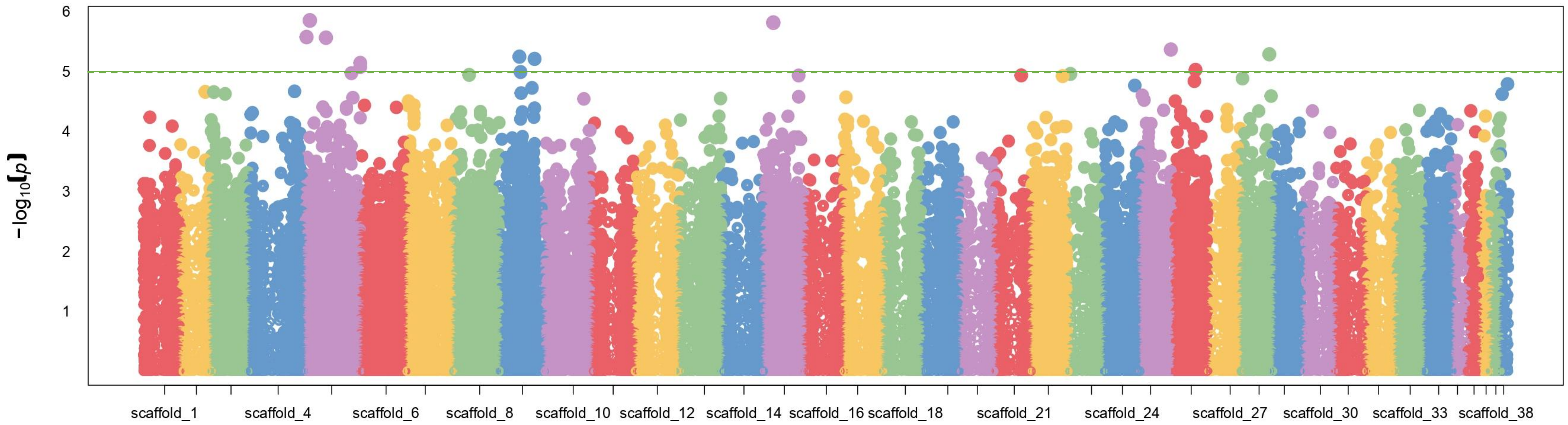

BLINK Acid detergent fiber (ADF) (Var 11)

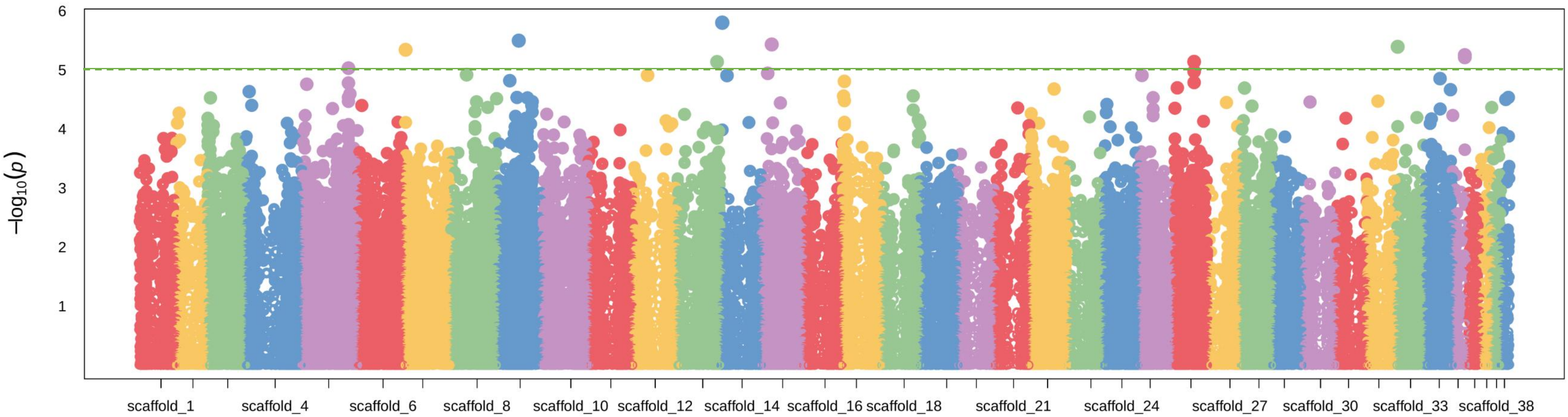

MLMM Acid detergent fiber (ADF) (Var 11)

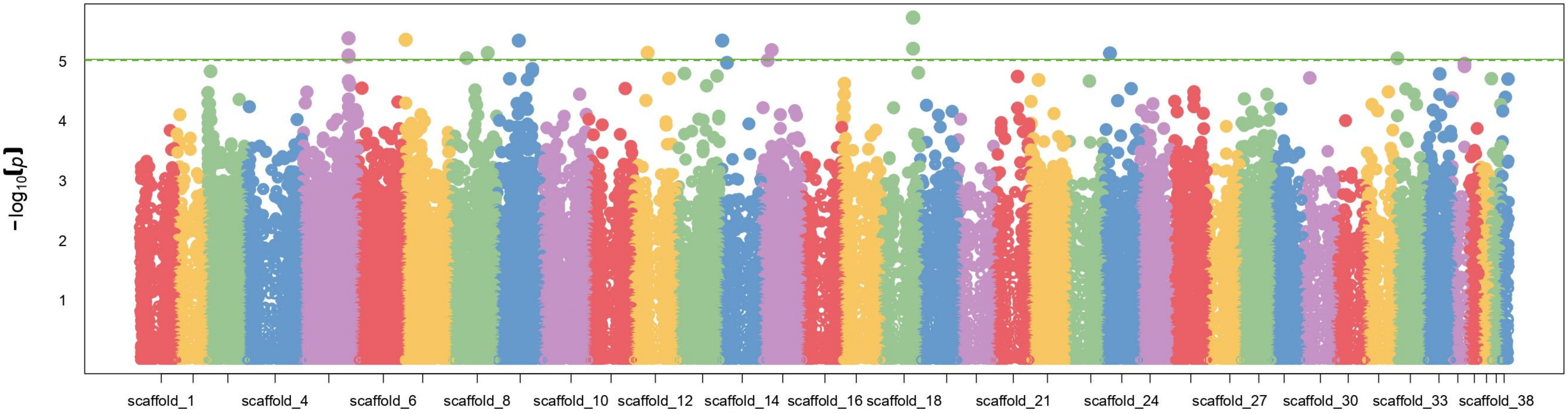

FarmCPU Acid detergent fiber (ADF) (Var 11)

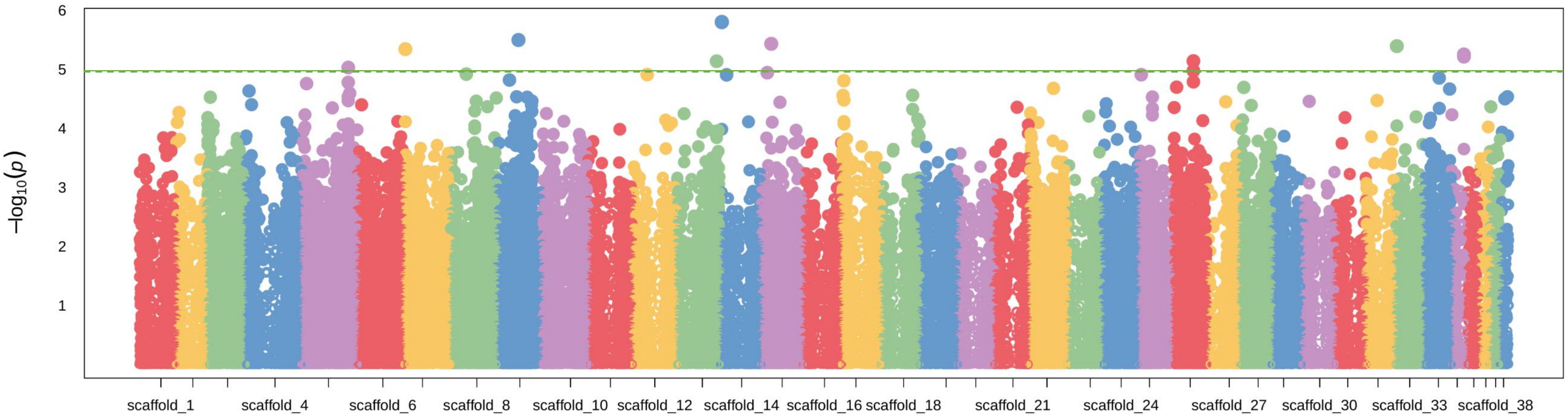

BLINK Neutral detergent fiber (NDF) (Var 12)

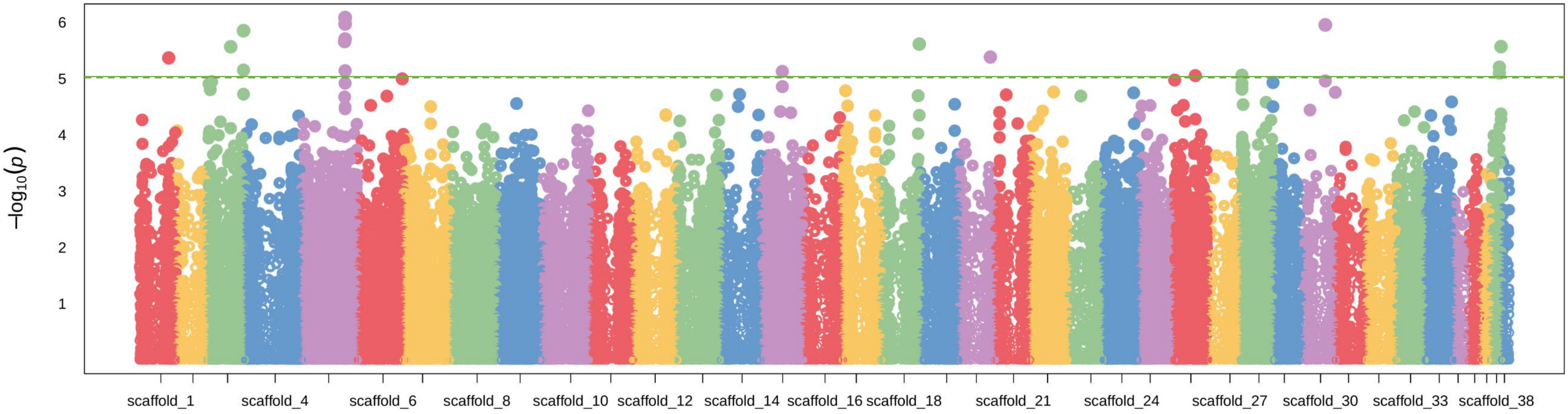

FarmCPU Neutral detergent fiber (NDF) (Var 12)

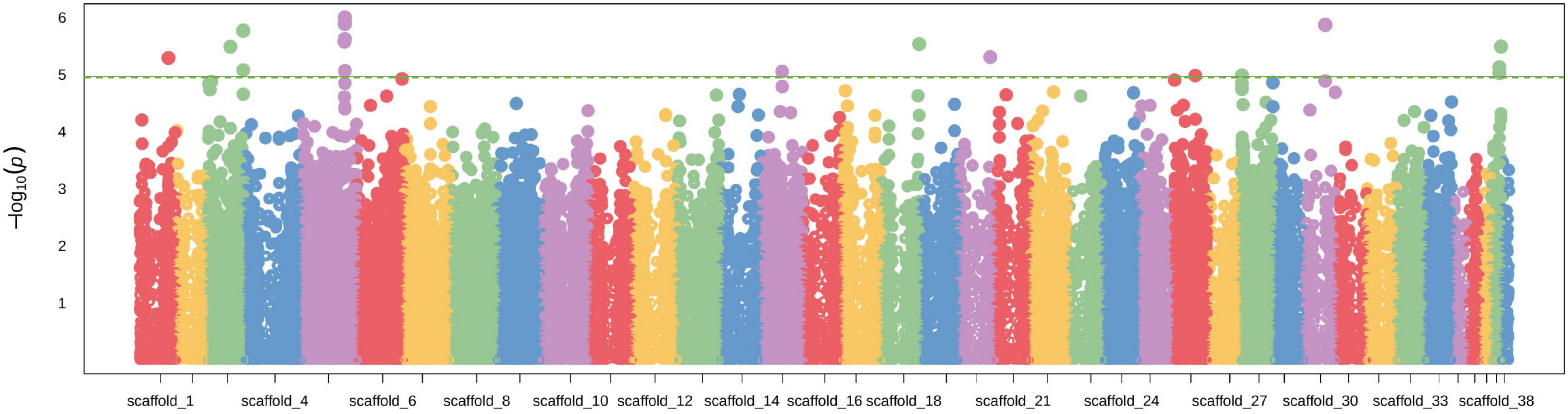

BLINK N uptake (Var 13)

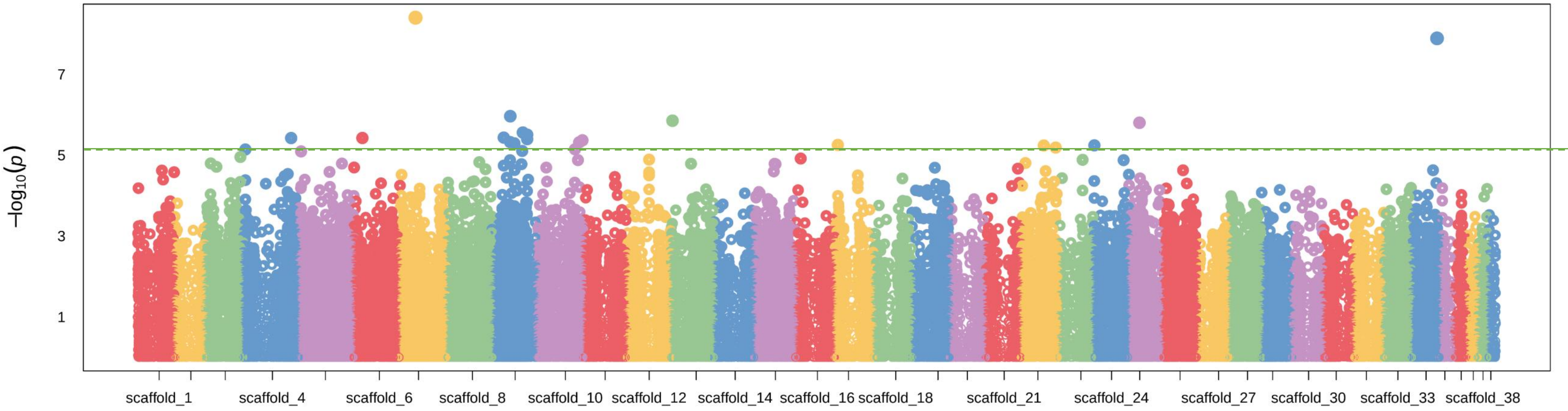

FarmCPU N uptake (Var 13)

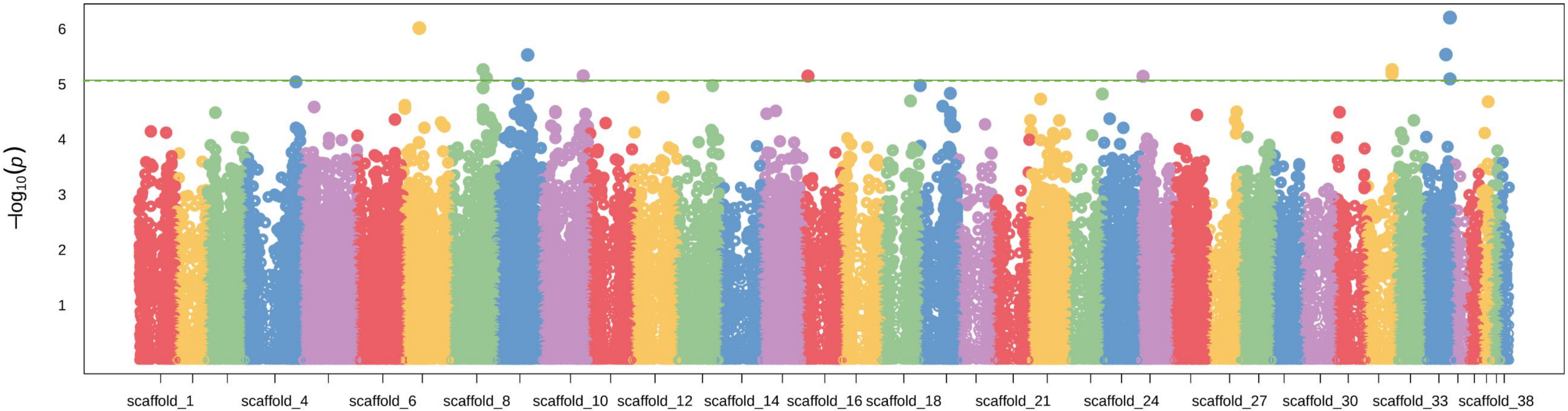

MLMM N uptake (Var 13)

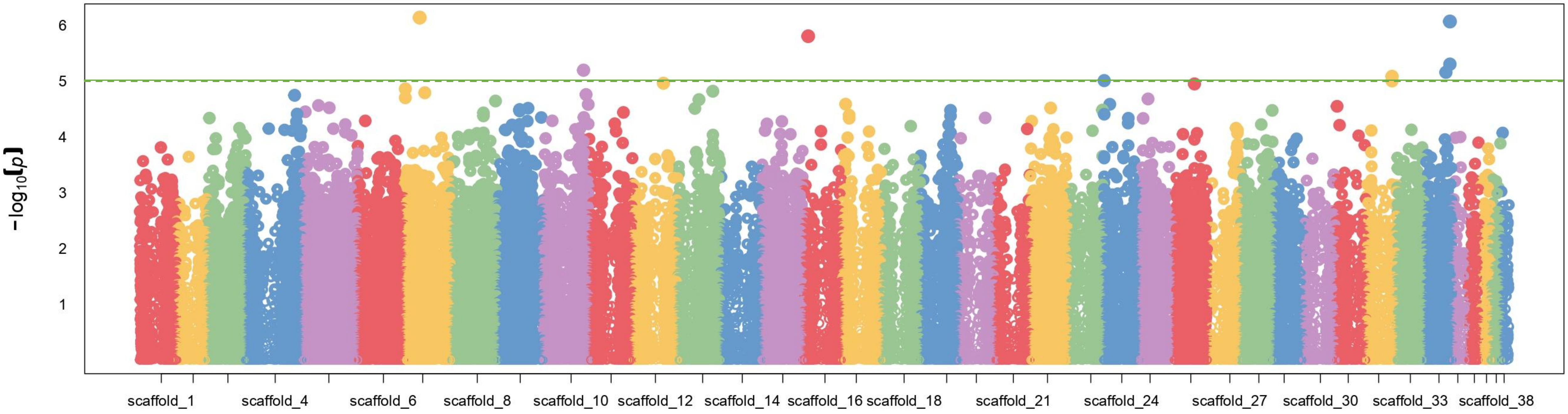

FarmCPU Nitrification Rate (Var 14)

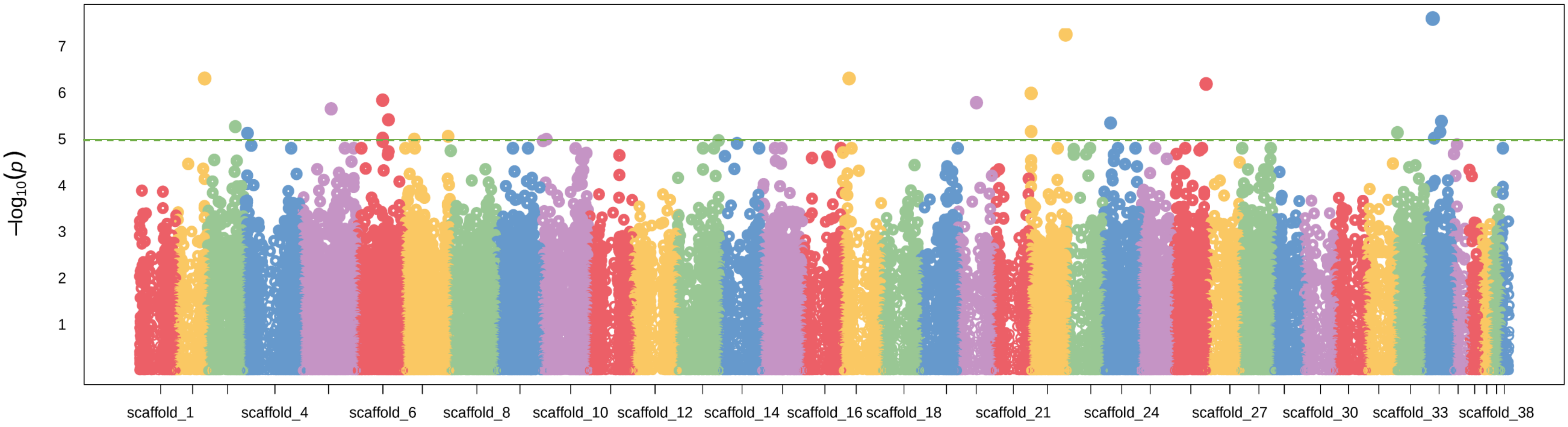

BLINK Nitrification Rate (Var 14)

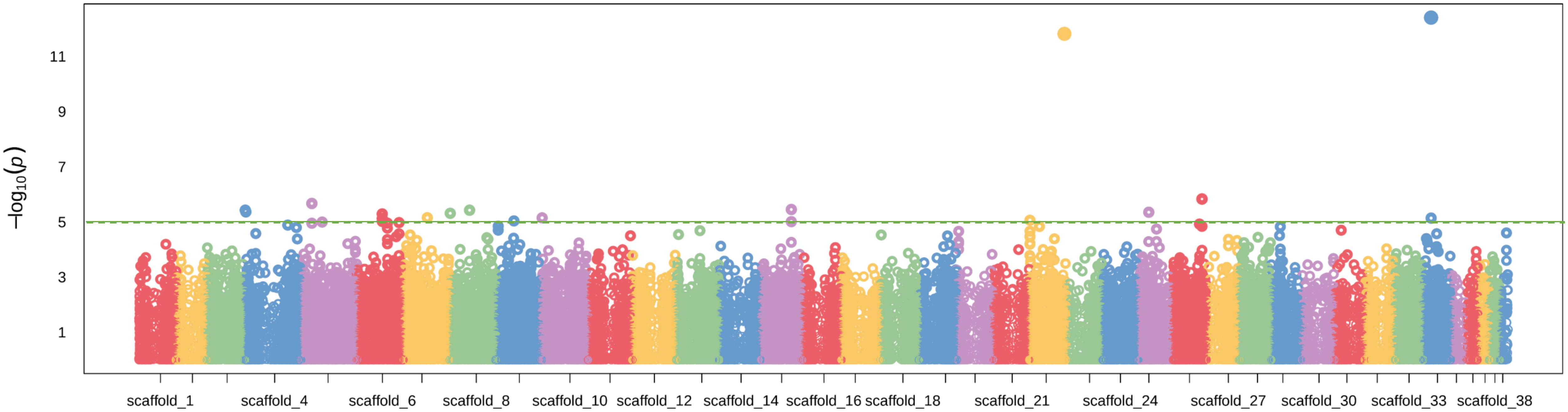

MLMM Nitrification Rate (Var 14)

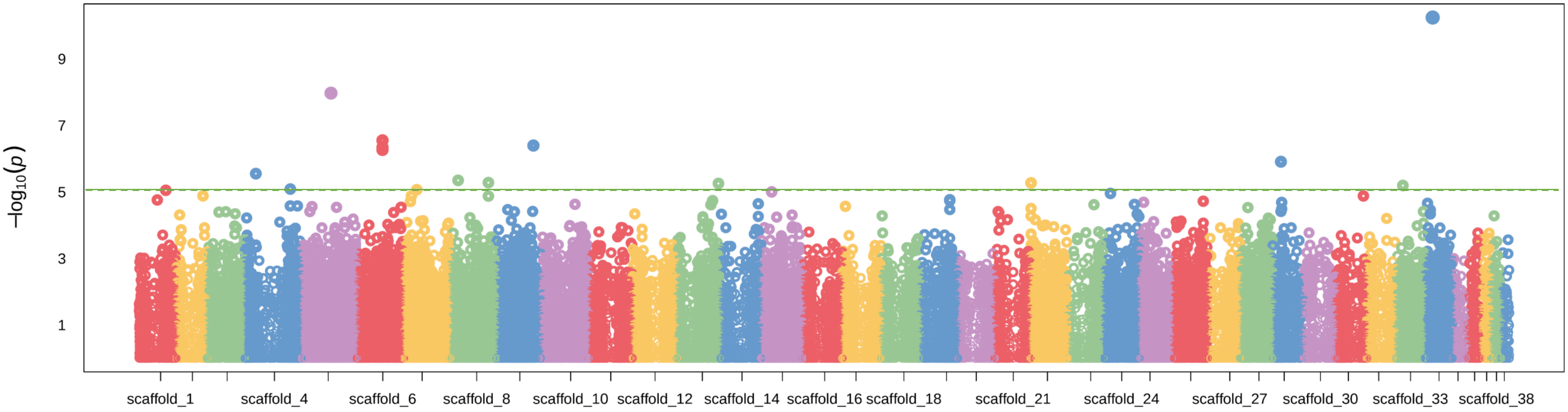

BLINK Shoot biomass production (SBP) (Var 15)

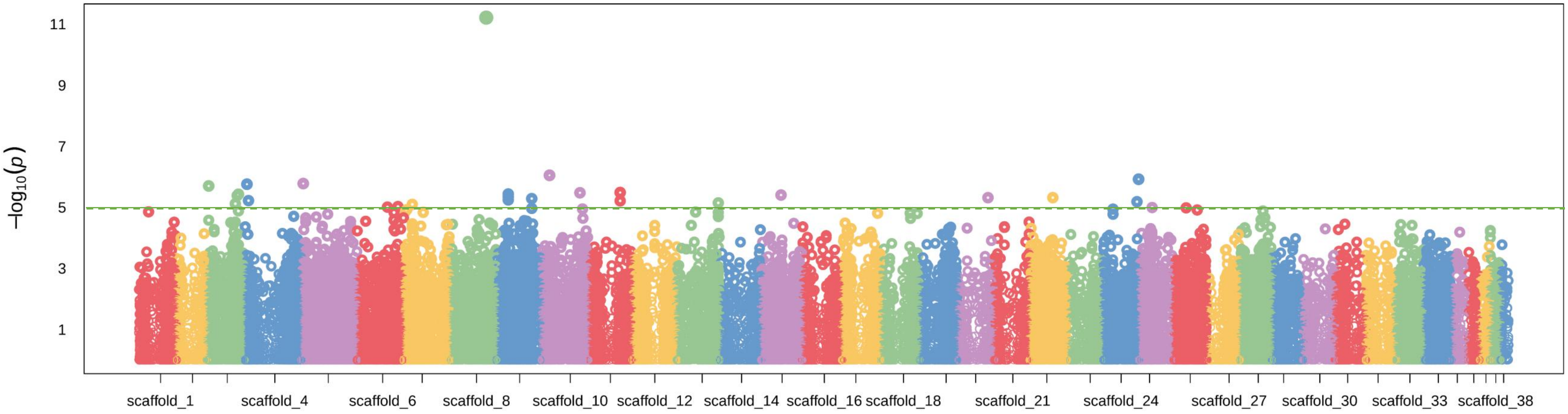

BLINK Green Forage Weight (GWF) (Var 16)

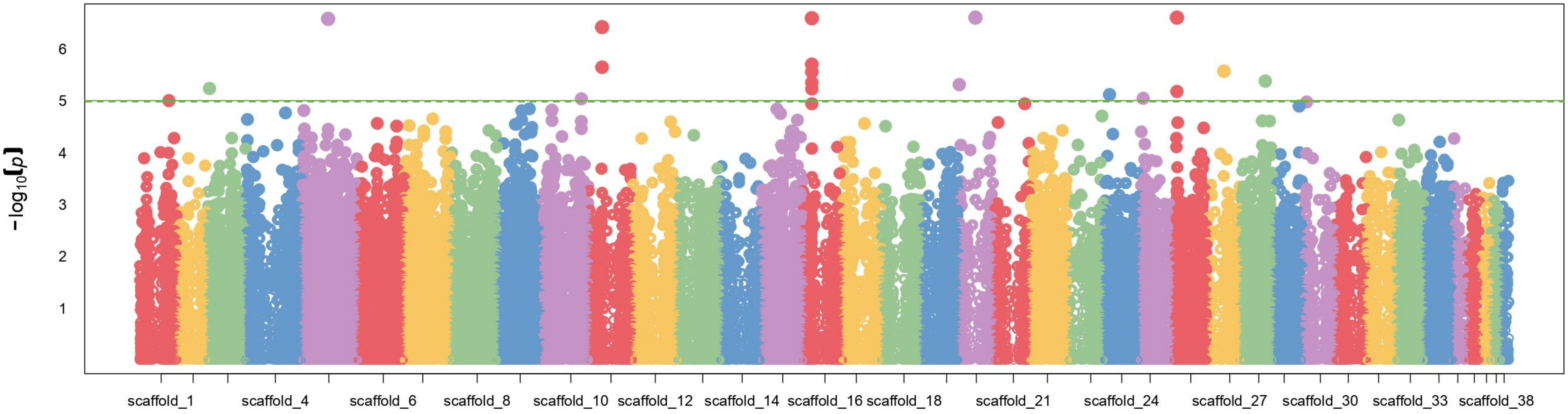

FarmCPU Green Forage Weight (GWF) (Var 16)

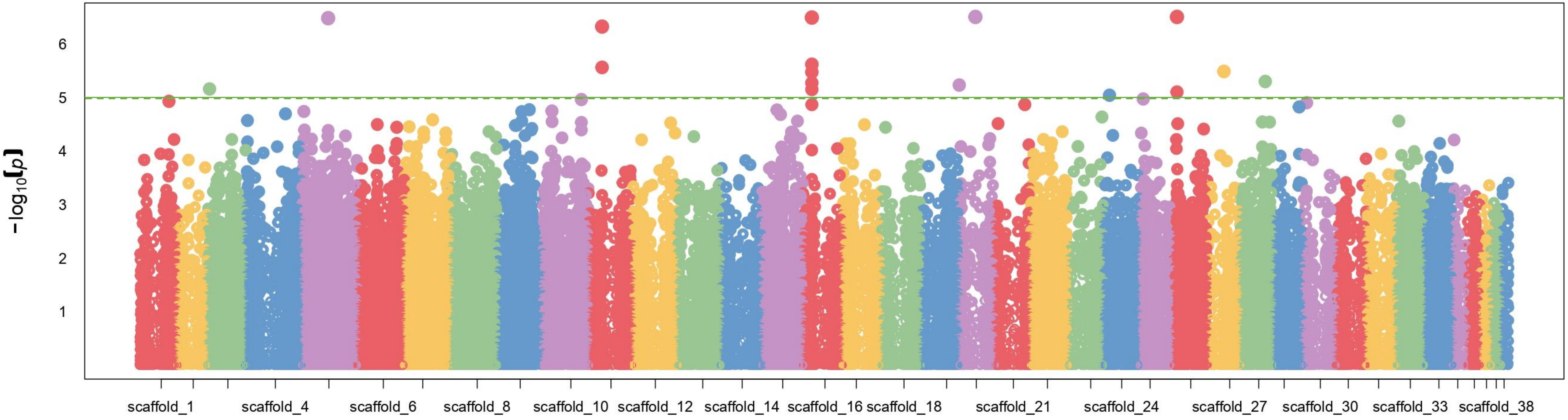

BLINK Green Forage Weight (GWF) (Var 17)

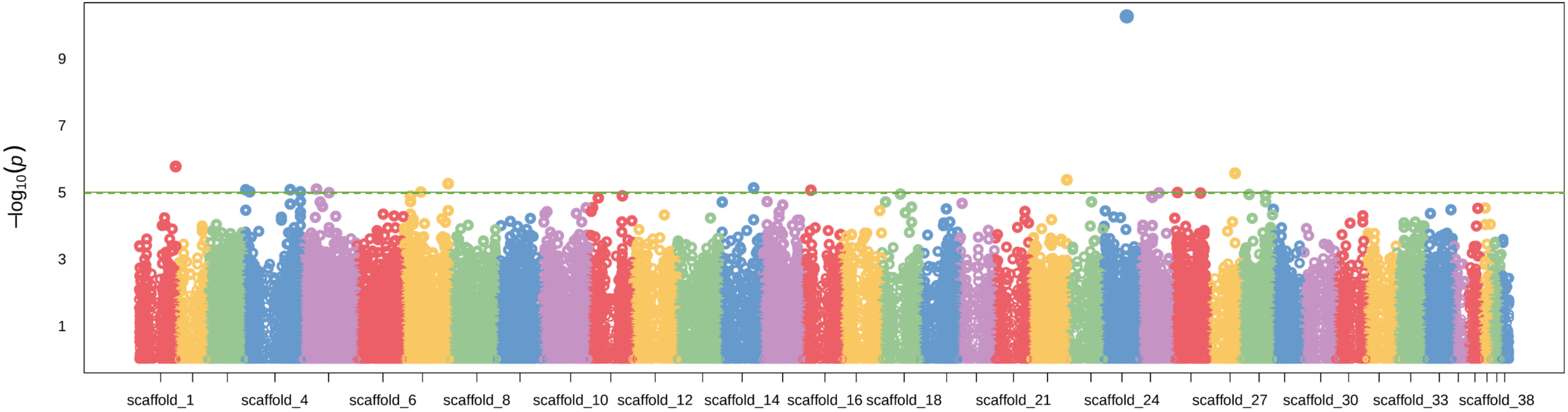

BLINK Green Forage Weight (GWF) (Var 18)

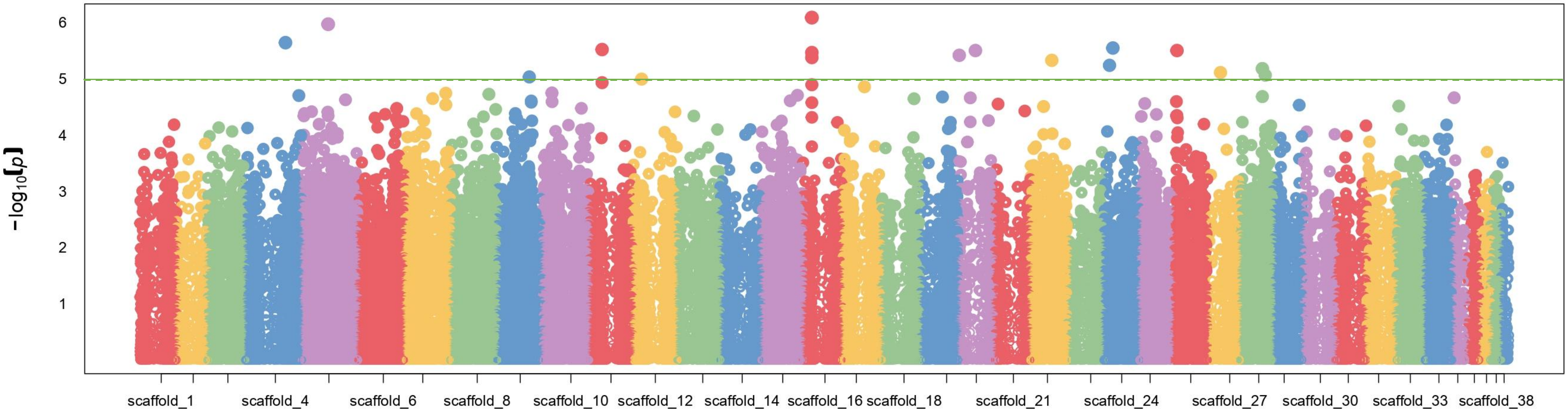

FarmCPU Green Forage Weight (GWF) (Var 18)

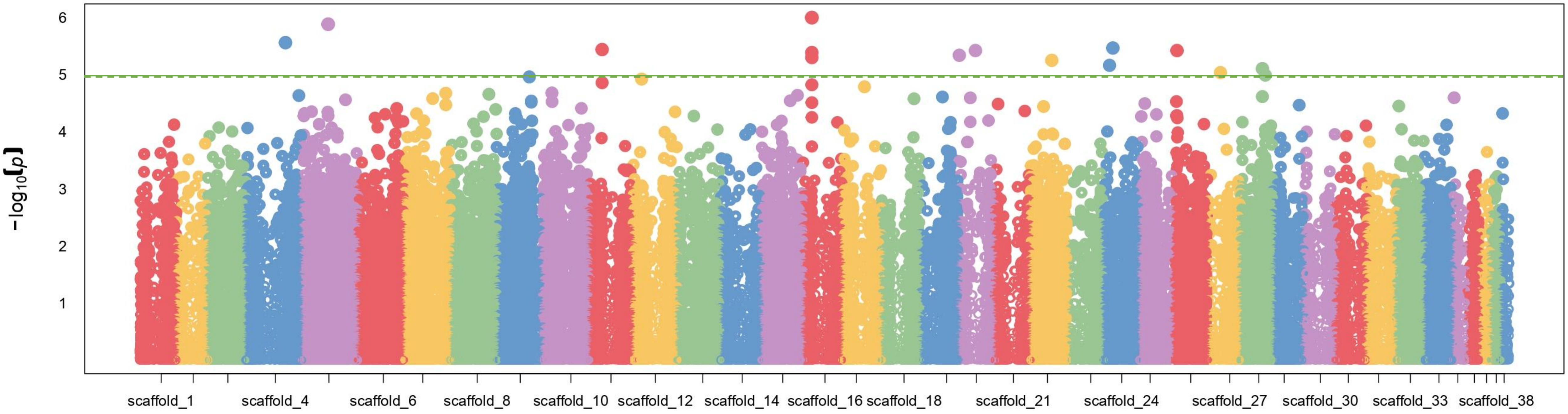

FarmCPU Dry Matter Yield (DMY) (Var 19)

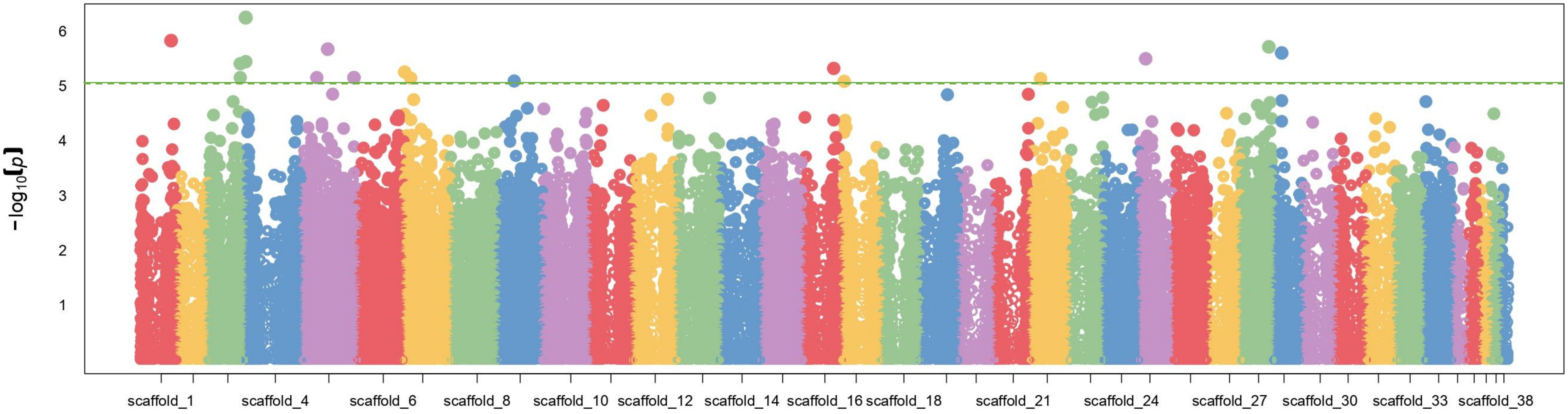

BLINK Dry Matter Yield (DMY) (Var 19)

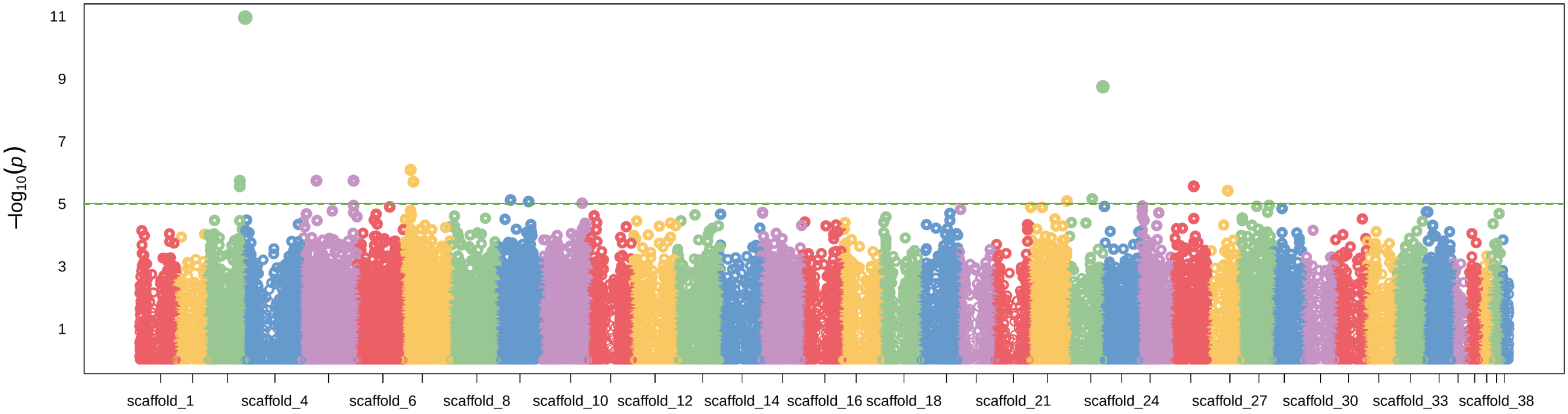

MLMM Dry Matter Yield (DMY) (Var 19)

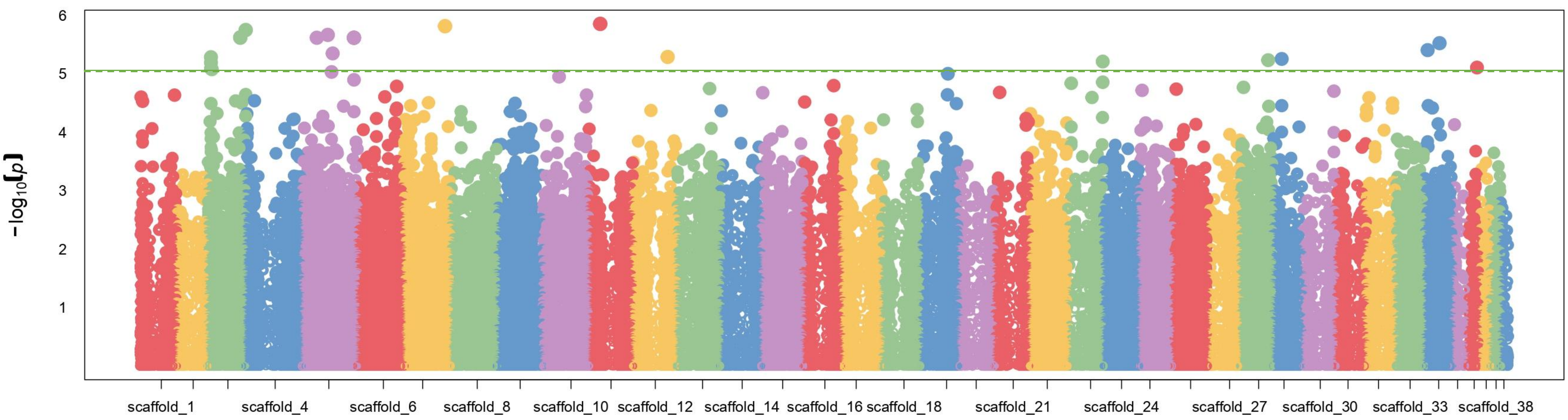

Supplement: Supplementary file 2 — Supplementary Material 2. [file 12870_2025_8007_MOESM2_ESM.pdf]
